# RNA-Seq Analysis Reveals MAPKKK Family Members Related to Drought Tolerance in Maize

**DOI:** 10.1371/journal.pone.0143128

**Published:** 2015-11-24

**Authors:** Ya Liu, Miaoyi Zhou, Zhaoxu Gao, Wen Ren, Fengling Yang, Hang He, Jiuran Zhao

**Affiliations:** 1 Maize Research Center, Beijing Academy of Agricultural and Forestry Science, Beijing 100097, P.R.China; 2 School of Life Sciences and School of Advanced Agriculture Sciences, Peking-Tsinghua Center for Life Sciences, Peking University, Beijing 100871, P.R.China; Estación Experimental del Zaidín (CSIC), SPAIN

## Abstract

The mitogen-activated protein kinase (MAPK) cascade is an evolutionarily conserved signal transduction pathway that is involved in plant development and stress responses. As the first component of this phosphorelay cascade, mitogen-activated protein kinase kinase kinases (MAPKKKs) act as adaptors linking upstream signaling steps to the core MAPK cascade to promote the appropriate cellular responses; however, the functions of MAPKKKs in maize are unclear. Here, we identified 71 MAPKKK genes, of which 14 were novel, based on a computational analysis of the maize (*Zea mays* L.) genome. Using an RNA-seq analysis in the leaf, stem and root of maize under well-watered and drought-stress conditions, we identified 5,866 differentially expressed genes (DEGs), including 8 MAPKKK genes responsive to drought stress. Many of the DEGs were enriched in processes such as drought stress, abiotic stimulus, oxidation-reduction, and metabolic processes. The other way round, DEGs involved in processes such as oxidation, photosynthesis, and starch, proline, ethylene, and salicylic acid metabolism were clearly co-expressed with the MAPKKK genes. Furthermore, a quantitative real-time PCR (qRT-PCR) analysis was performed to assess the relative expression levels of MAPKKKs. Correlation analysis revealed that there was a significant correlation between expression levels of two MAPKKKs and relative biomass responsive to drought in 8 inbred lines. Our results indicate that MAPKKKs may have important regulatory functions in drought tolerance in maize.

## Introduction

The world population is increasing at an alarming rate, while abiotic stresses play crucial roles in crop failures and reductions in field crop productivity. Drought is the most important environmental stress affecting agricultural production. Maize *(Zea mays* L.), which is frequently exposed to drought stress conditions, is one of the most important cereal crops in the world, together with rice and wheat. In recent years, some studies have found that several genes encoding protein kinases can activate responses to abiotic stresses, such as drought [1, 2]. Drought stress is one of the major limiting factors for maize production. Some studies have revealed that drought stress can affect maize, especially during the reproductive stage. Their working hypothesis involves signaling events associated with increased ABA levels, decreased glucose levels, the disruption of ABA/sugar signaling, and the activation of programmed cell death/senescence through the repression of a phospholipase C-mediated signaling pathway [3]. A total of 524 non-synonymous single nucleotide polymorphisms (nsSNPs) that were associated with 271 candidate genes for drought tolerance involved in plant hormone regulation, carbohydrate and sugar metabolism, signaling molecules regulation, redox reaction and acclimation of photosynthesis to environment were detected by common variants (CV) and cluster analyses with the availability of maize B73 reference genome and whole-genome resequencing of 15 maize inbred lines [4]. These selected genes will not only facilitate our understanding of the genetic basis of the drought stress response but will also accelerate genetic improvement through marker-assisted selection in maize.

The mechanisms of drought tolerance in plants are complex. Effects of drought on plant hormone signal transduction, protein modification and photosynthesis have been reported [1–3, 5]. It has been demonstrated that the accumulation of proline can increase tolerance to water stress in plants. Transgenic plants with increased proline content produce higher biomass under drought [6]. An analysis of *edr1* mutation determined that the *EDR1* gene functions in negative regulation after salicylic acid treatment [7]. The stress-induced increase in reactive oxygen species (ROS) in plant cells results from an imbalance between generation and degradation. Drought stress can successively induce stomatal closure, moderate increases in ROS and decreases in photosynthesis. As described above, the response of plants to drought stress involves numerous genes and pathways related to diverse mechanisms.

Some molecular and biochemical studies have revealed that drought stress can elicit defense responses through MAP kinase pathways [8]. For example, a functional analysis of Arabidopsis demonstrated that the expression of *AtMPK3*, which shows high sequence similarity to *WIPK*, was increased when plants underwent drought [9]. The expression of *OsMAPK5*, *OsMSRMK2*, *OsMAPKK44* and *OsMKK1* was inducible by drought treatment in rice [10–13]. An analysis of transgenic rice plants strongly revealed that OsMAPK5 could regulate drought tolerance. Moreover, in maize, the transcript level of *ZmMPK3* was increased by drought stress [14]. An investigation in alfalfa indicated that MKK4 kinase is transiently induced by drought treatment [15]. In addition, it has been reported that the tomato MAPK gene *SIMPK4*, which shows homology with Arabidopsis *AtMPK4*, can improve tolerance to drought stress [16]. In this study, we investigated the relationship between MAPKKK genes and drought stress in maize. Several previous studies have indicated that MAPKKK genes can be activated by drought treatment. In Arabidopsis, the mRNA level of the MAPKK kinase AtMEKK1, which can activate its downstream factors ATMKK2 and MEK1, can be increased by drought [9, 17]. A MAPKKK gene that responds to drought has also been identified in rice. Furthermore, NPK1 isolated from tobacco [18] has been reported to positively regulate drought tolerance. *NPK1* transgenic maize and rice both have higher yields under drought conditions compared with their non-transgenic counterparts [19–21].

The mitogen-activated protein kinase (MAPK) cascade, which is highly conserved, is a major signal transduction pathway in all eukaryotes, including yeasts, animals and plants [22]. The MAPK signaling pathway, which plays a pivotal role in plant cellular responses, is involved in cell division, differentiation, apoptosis, and responses to a diversity of environmental stimuli, including cold, heat, drought and pathogen attacks [17, 23–34]. MAPK, which consist of MAPKs, MAP kinase kinases and MAP kinase kinase kinases, are serine/threonine-specific protein kinases. To transduce external stimuli into cellular responses, MAPK cascades transfer these signals via phosphorylation [22, 25, 33]. By scanning the Arabidopsis genome, 80 MAPKKKs, 10 MAPKKs and 20 MAPKs have been identified [25, 35]. Previous analyses of the rice genome have identified 75 MAPKKKs, 8 MAPKKs and 17 MAPKs [36, 37]. Currently, 9 MAPKKs and 19 MAPKs have been characterized in maize [38–45]. In plants, MAPKKKs have been divided into three groups, the MEKK-like family, the Raf-like family and the ZIK-like family [30, 35, 46–50]. Among these groups, MEKK-like family is most similar to animal MEKKs and yeast MAPKKKs. Most of the Raf family proteins have a C-terminal kinase domain and extend N-terminal regulatory domain. Whereas most of the ZIK family members have N-terminal kinase domain and members of MEKK family has less conserved protein structure with kinase domain located either at N- or C-terminal or central part of the protein [37].

In addition, it is well documented that MAPKKKs are related to many stress-response pathways, such as plant hormone signal transduction and oxidative signaling. The Raf-like MAPKKK gene in rice, *DSM1*, functions as an early signaling component to regulate the response to drought stress through ROS (reactive oxygen species) scavenging [51]. The results were identified by examining *dsm1* mutants. *DSM1*-RNA interference lines were hypersensitive to drought stress and were more sensitive to oxidative stress. *DSM1* may also function in the drought stress signaling response through an ABA-independent pathway that involves oxidative stress signaling [51]. Moreover, *ANP1* has been shown to be involved in auxin signaling transduction and the oxidative stress signaling pathway [52, 53]. In turn, oxidative stress can activate MAPK cascades to mediate the induction of oxidative stress-responsive genes. The overexpression of *ZmMKK1* in Arabidopsis can enhance drought tolerance and enhance the expression of ROS-scavenging enzyme-related genes [54]. Analyses in maize have revealed the involvement of *ZmMPK3* and *ZmMPK5* in ROS signaling pathways [14, 55]. Data from recent studies provide evidence that *ZmMKK1*, *ZmMKK3* and *ZmMKK4* can enhance drought tolerance through ROS scavenging [38, 54, 56]. Additionally, antioxidant enzyme activities can be enhanced in ZmMKK4-overexpressing plants compared with wild-type plants [56]. In brief, MAPK cascades can respond to drought stress through different pathways. However, the involvement of MAPKKKs in the drought response in maize is not yet clear.

In maize, 74 MAPKKKs had been reported by searching against the maize genome database and NCBI[57]. In this work, we will search against the maize B73 genome in the MaizeGDB to identify MAPKKK genes in maize using the BLAT program with a rigorous limiting condition. To further explore whether the identified maize MAPKKKs are related to drought stress, a variety of maize grown widely in SouthWest China, ZD619, and eight maize inbred lines were used in the RNA-seq, qRT-PCR and biomass analyses respectively. Additionally, important metabolic processes and regulatory process pathways related to the identified MAPKKK genes were analyzed to investigate the MAPKKKs involved in the drought stress response in maize.

## Materials and Methods

### Plant materials and stress treatments

In this experiment, the RNA-seq used ZD619, a variety of growing widely in SouthWest China, which showed strong drought resistance in the field. Eight inbred lines consisting of J24, J853, X178, Q319, B73, E28, C8605-2 and 200B as well as ZD619 were used to further analyze on their expression pattern by qRT-PCR and phynotype discrimination responsive to drought stress. The maize seeds were pre-germinated in the plant incubator. The uniformly germinated seeds were chosen and sown in plastic pots (23cm×16 cm) which filled with a 1:1:1 mix of soil:vermiculite:nutrient soil in greenhouse under 16h of light (25°C) and 8h of dark (20°C). Four uniformly strong plants finally retained at the 2–3 leaf stage.

The experiment included control (80% of relative soil water content, RSWC) and drought stress (35% of RSWC). Each treatment was comprised of two replications. The RSWC for the control plants exceeded to 80%. The drought treatment plants did not receive water from three-week-old seedling until the RSWC decreased to 35%, the drought treatment takes about one week, then kept stable for 2–3 days.

### Plant biomass determination

Each plant except for qRT-PCR analysis was rapidly collected and weighed for the fresh biomass of two replicates in the greenhouse. Then the entire plant was put into a paper packet and was dried at approximately 75°C in a drying cabinet. The dry biomass was determined until 48 hours.

### RNA isolation and qRT-PCR

The samples were collected and were immediately frozen in liquid N_2_ for further use. Each total RNA sample of three tissues, leaf (the top three leaves), stem and root, was extracted from four uniformly plants using an RNeasy Plant Mini Kit followed by RNase-Free DNase Set (Qiagen, Hilden, Germany). The extracted RNA was assessed by a NanoDrop 2000 Spectrophotometer, 1% agarose gel electrophoresis. The poly(A) mRNA was isolated from purified total RNA using oligo(dT) magnetic beads. The mRNA was fragmented into short pieces by adding the fragmentation buffer. The first-strand cDNA was synthesized by reverse transcriptase and random primers using mRNA fragments as templates. Second-strand cDNA synthesis was produced by RNase H and DNA polymerase I. Double-stranded cDNA fragments went through end repair, 3’dA tailing and adapter ligation. The required fragments were then purified and enriched by PCR to create the final cDNA library. The sequences were generated by Illumina HiSeq 2000 sequencer.

In qRT-PCR three biological replicates were conducted and each biological replicate was technically repeated three times. The qRT-PCR was carried out using Maxima SYBR Green/ROX qPCR Master Mix (2×) (Thermo Scientific) performed with a 7300 Real-Time PCR System (Applied Biosystems, Foster City, CA) according to the supplier's protocols. Each PCR reaction mixture contained 12.5μl of 2×real-time PCR mix, 1μl of gene-specific primers, 1μl reverse transcribed cDNA product and water. The thermal cycle applied was as follows: 95°C for 10min followed by 40 cycles of 95°C for 15s, 60°C for 30s, 72°C for 30s, 82°C for 5s. At the end of the PCR cycles, melting curve analysis was performed using a single cycle consisting of 95°C for 15s and 60°C for 1min followed by a slow temperature increase to 95°C at the rate of 0.3°C/s. Relative gene expression was calculated according to the delta-delta Ct method of the system.

### Definition of the maize MAPKKKs and phylogenetic analysis

Maize protein sequences were downloaded from MaizeGDB database (http://www.maizegdb.org/). The maize MAPKKKs was identified through BLAT searches against Arabidopsis, rice MAPKKK query sequences [25, 35, 37]. 25% identity was taken as the threshold, and the query sequences which aligned 5 BLAST alignments passed the threshold in MAPKKK gene family in Arabidopsis and rice were collected as MAPKKKs in maize.

Multiple sequence alignments were conducted on the amino acid sequences of MAPKKK proteins in maize, Arabidopsis and rice genomes using ClustalW with default settings. Phylogenetic tree was constructed by MEGA5.0 software based on alignments using Maximum Likelihood method with 1000 bootstraps to investigate the evolutionary relationship among MAPKKK proteins in three species. Analyze the conserved domain of the full protein sequences of MAPKKK genes by BioEdit.

### Subcellular localization and chromosomal locations

The predicted subcellular localization of maize MAPKKKs was retrieved using the CELLO v2.5 server (http://cello.life.nctu.edu.tw/). Information about the physical locations of maize MAPKKK genes on chromosomes was obtained from MaizeGDB database. The chromosomal locations of the MAPKKK genes in maize were mapped on chromosomes using PERL script and Adobe Illustrator Artwork software.

### RNA-seq analysis and identification of DEGs

The RNA-seq reads for each tissue were mapped to maize reference genome B73 using the Tophat [58]. Then the differentially expressed genes between drought processed sample and well-water processed sample in three tissues were extracted after processing Cufflinks and Cuffdiff (http://cufflinks.cbcb.umd.edu/manual.html).

We identified differentially expressed genes (DEGs) by fold change greater than two and p-value≤0.05.

Our raw data and the processed RNA-seq data have been deposited in the National Center for Biotechnology Information Gene Expression Omnibus (GSE71377).

### GO Analysis and Functional classification

We used an online tool Venn (http://bioinfogp.cnb.csic.es/tools/venny/index.html) to compare the DEGs in three tissues. The cluster was done by cluster3.0 using the Complete linkage method. The heatmap was drew by treeview and we chose the log2 (fold change) absolute value one as scale. Functional classification was performed by using the tool agriGO (http://bioinfo.cau.edu.cn/agriGO/). The functional clusters enrichment analysis was calculated by comparing with the whole maize genome V5a by Singular Enrichment Analysis (SEA) method, and the highest classification stringency was chosen for clustering by FDR≤0.05.

We used an interactive ontology tool PageMan to generate overview graphs for profiling experiments with maize database and a user-driven tool MAPMAN to display genomics data sets onto diagrams of metabolic pathways and other biological processes. The Wilcoxon test likes a t-test was used to test the hypothesis that objects within one functional class behave differently from the rest of the objects. The significance of the change is reflected in the intensity and the direction by the color. The number used to do the heatmap was calculated by p-value. All p-values above 0.05 are set to a z-score of 0 to avoid misinterpretation. A highly saturated color indicates a high absolute value, whereas smaller values are indicated by lower color saturation. The two different colors can be selected to distinguish between categories where the average of the signals for all the genes in a category increases (red) or decreases (blue).

### Co-expression analysis

We carried out pathway enrichment analysis by KAAS (http://www.genome.jp/tools/kaas/) for our DEGs in three tissues with Arabidopsis database. 14 common regulated pathways and 5 tissue specific regulated pathways were used for the heat map show. An R package WGCNA was used for our sequenced maize transcripts. We kept the transcripts by at least FPKM>0.1 in six samples. Firstly, we obtained the DEGs in each selected pathways and the co-expressed MAPKKKs and the co-expression score between each two genes. Secondly, the cutoff of 0.2 was used to select co-expression level in all the three tissues and a standard Z-score for each term is given by the following formula: $Z=(Sm-\mu )\times \sqrt{m}/\delta$, where Sm is the mean co-expression value for genes selected with the cutoff, μ is the mean co-expression value of the entire gene list from WGCNA, m is the number of MAPKKK genes co-expressed with the DEGs, and δ is the SD of the co-expression value in the entire gene list [3]. A cutoff value of 0.2 was used to select highly co-expression events.

### Genes specific primers

First-strand cDNA was used as a template in a qRT-PCR synthesized from 5μg total RNA using the SuperScript III First-Strand Synthesis System for RT-PCR (Invitrogen Corp.). The endogenous reference zSSIIb from *zea mays* starch synthase II was used as an internal control to normalize the data. The eight genes specific primers which designed based on the cDNA sequences were used to detect the gene expression level. The qRT-PCR primers were check the specification by NCBI primer-BLAST and validated by PCR(S4 File).

## Results

### Physical location and phylogenetic analysis of MAPKKKs in maize

**Fig 1:**
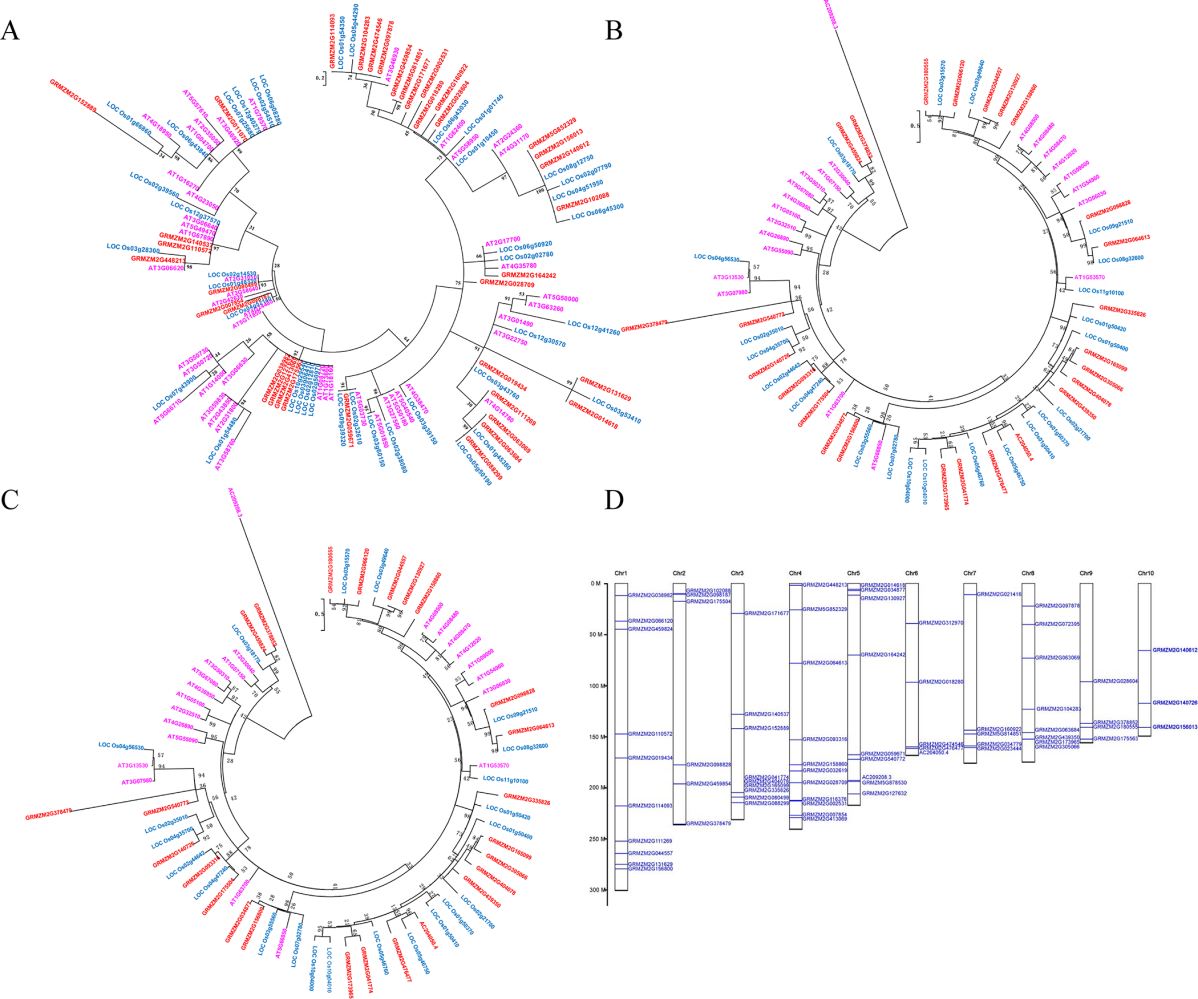
Phylogenetic tree and genomic locations of maize, rice and Arabidopsis MAPKKK family genes. The different colors represent three species. Red represents maize, blue represents rice, and pink represents Arabidopsis. (a). Raf subfamily. (b). MEKK subfamily. (c). ZIK subfamily. (d). Physical locations of MAPKKK genes on maize chromosomes.

To identify MAPKKK genes in maize, the Arabidopsis and rice MAPKKK protein sequences were employed to perform a BLAST search against the protein sequences of maize B73. In maize, 71 MAPKKKs were identified, including 57 reported MAPKKKs [57] and 14 novel ones (Table A in S1 File), which were basically consistent with previous research. These reported MAPKKKs were named according to the foregoing study [57], while since there was no standard nomenclature followed for MAPKKKs neither in Arabidopsis nor in rice, the 14 putative MAPKKKs were designated according to their group (Table A in S1 File).We obtained the chromosomal locations of these 71 MAPKKKs, and the predicted subcellular localization was determined from the CELLO v2.5 server (Table A in S1 File). The physical location data showed that 70 MAPKKKs were distributed on all 10 maize chromosomes (Fig 1D), except the *GRMZM2G011070* (*ZmMAPKKK29*) gene, which was situated on an undetermined chromosome (chromosome unknown). Among the 14 novel MAPKKKs, *GRMZM2G459824* (*ZmMAPKKK75*) was located on chromosome 1; *GRMZM2G404078* (*ZmMAPKKK76*) and *GRMZM2G335826* (*ZmMAPKKK77*) were located on chromosome 3; and *GRMZM2G158860* (*ZmMAPKKK78*), *GRMZM2G032619* (*ZmMAPKKK81*) *GRMZM2G002531* (*ZmMAPKKK85*), *GRMZM2G028709* (*ZmMAPKKK86*) and *GRMZM5G852329* (*ZmMAPKKK87*) were located on chromosome 4. *GRMZM2G127632* (*ZmMAPKKK88*) was located on chromosome 5. Chromosome 6 contained *AC204050*.4 (*ZmMAPKKK79*). Chromosome 7 contained *GRMZM2G034779* (*ZmMAPKKK82*) and *GRMZM2G023444* (*ZmMAPKKK83*). *GRMZM2G072395* (*ZmMAPKKK84*) was located on chromosome 8. *GRMZM2G378852* (*ZmMAPKKK80*) was located on chromosome 9.

The phylogenetic analysis was performed using the protein sequences in Arabidopsis, rice and maize. We aligned the full protein sequences of all the MAPKKKs in Arabidopsis, rice and maize using ClustalW, and we built a phylogenetic tree using MEGA5.0 (Fig 1). We identified 80 MAPKKKs in Arabidopsis and 75 MAPKKKs in rice [25, 35, 37], which could be subdivided into three major subtypes, Raf, MEKK and ZIK [30]. Based on the subtypes subdivided in a recent analysis, the MAPKKKs in maize could also be divided into three major groups, including Raf, MEKK and ZIK. The MEKK group included 26 MAPKKKs in maize, 22 MAPKKKs in rice, and 21 MAPKKKs in Arabidopsis. The ZIK group included 8 MAPKKKs in maize, 10 MAPKKKs in rice and 11 MAPKKKs in Arabidopsis. The Raf group included 37 maize MAPKKKs, 43 rice MAPKKKs and 48 Arabidopsis MAPKKKs. As the results indicated, MAPKKKs are highly conserved among plants. Furthermore, the degree of conservation in the three subfamilies was also very high, and the numbers were similar in species that may have similar functions. Similar to the conservation of the MAPKKK motif in Arabidopsis and rice, the ZIK family in maize shared the conserved domain GTPEFMAPE (L/V) (Y/F) (Fig A(a) in S5 File), and the MEKK family in maize had the conserved domain G (T/S) Px (W/F) MAPEV (Fig A(b) in S5 File). Furthermore, the conserved domain GTxx (W/Y) MAPE was detected in the Raf family in maize (Fig A(c) in S5 File).

### RNA-seq analysis of well-watered and drought-stressed maize transcriptomes in three tissues

**Fig 2:**
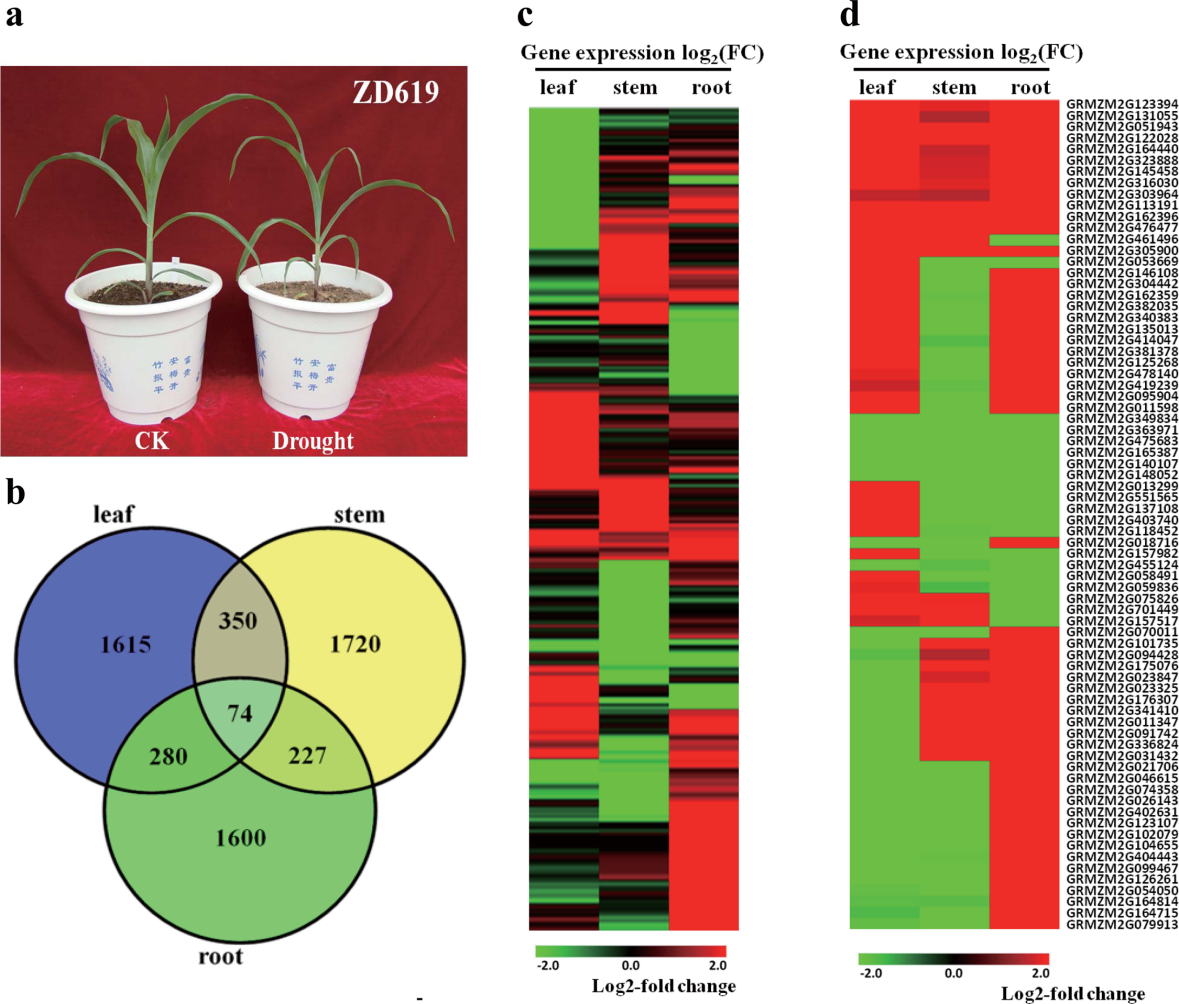
Differentially expressed genes in leaf, stem, and root based on RNA-seq data. (a). The growth phenotypes of ZD619 in the eight leaf stage under drought and well-water conditions. The well water-treated plant was shown on the left and drought-treated plant was shown on the right in the picture. (b). Venn diagram of DEGs in leaf, stem, and root. (c). Heat map of 5,866 DEGs in the three tissues. The genes in at least one of the subsets were analyzed. The bar represents the log2 of the drought/control ratio. (d). Heat map of 74 DEGs in the three tissues. The bar represents the log2 of the drought/control ratio.

The maize variety ZD619 was selected to analyze differentially expressed genes in response to drought stress (Fig 2A). RNA samples from leaf, root and stem tissues of ZD619 under well-watered and drought stress conditions were isolated for next-generation sequencing using the Illumina HiSeq 2000 platform. The raw data included ~40 million reads from each sample. The quality of the sequencing data is very well. The Q20 which represents for the percentage of the base whose quality is greater than 20 is approximately 98%. And the Q30 can also be around 93%. (Table B in S1 File) After trimming the adaptor and low-quality reads, approximately 85% of the processed data could be aligned to the B73 reference using TopHat [58] (Table C in S1 File). Then, using Cuffdiff, 5,866 differentially expressed genes (DEGs) were identified in leaf, stem and root (S2 File). Among them, 2,319 DEGs were identified in leaf, including 1,206 that were up-regulated and 1,113 that were down-regulated. 2,371 DEGs were identified in stem, including 1,057 that were up-regulated and 1,314 that were down-regulated. 2,181 DEGs were also identified in root, including 1,544 that were up-regulated and 637 that were down-regulated (Table 1, S3 File, Fig C in S5 File).

**Table 1:** Differentially expressed genes (drought vs. control).

Tissue	Up-regulated	Down-regulated
leaf	1,206	1,113
stem	1,057	1,314
root	1,544	637

Through comparing the DEGs in leaf, stem and root, we found that only 74 genes were commonly regulated in all three tissues in response to drought, which suggested that these genes may play key roles during drought stress and that most of these genes were tissue-specific (Fig 2B). The heat map of the DEGs also demonstrated significant differences among the three tissues (Fig 2C). Out of those genes, there are 74 genes commonly regulated in the three tissues, approximately half of them were oppositely regulated in different tissues, which also indicated a large difference in the manner of regulation in different tissues (Fig 2D). Among the 74 highly significant co-regulated genes, *GRMZM2G476477* (*ZmMAPKKK20*) is a member of the MAPKKKs, which showed significant up-regulation in all three tissues. In addition, it has been reported that *GRMZM053669*, *GRMZM011598* and *GRMZM059836* were up-regulated under dehydration conditions [59]. *GRMZM2G051943* is a gene related to plant defense [60]. *GRMZM2G162359* is a gene related to *Aspergillus flavus* pathogenesis [61]. *GRMZM2G137108* and *GRMZM2G455124* are required for leaf development [62]. *GRMZM2G419239* and *GRMZM2G341410* are involved in cell wall-related biogenesis [63].

In maize, 9 MAPKKs and 19 MAPKs have been identified [38–45]. Among the 5,866 DEGs, *GRMZM2G344388* (ZmMKK10-2) is a member of MAPKK family[45], *GRMZM2G053987* (ZmMPK3-1) and *GRMZM2G062761* (ZmMPK15) are two members of MAPK family[44]. However, ZmMKK10-2 had no interaction with ZmMPK3-1 or ZmMPK15 [45]. ZmMKK10-2, ZmMPK3-1 and ZmMPK15 were up-regulated in leaf and stem but down-regulated in root under drought conditions. Significantly, we have found other highlighted members of the cascade, MAPKK and MAPK differentially expressed in their transcriptome levels.

**Fig 3:**
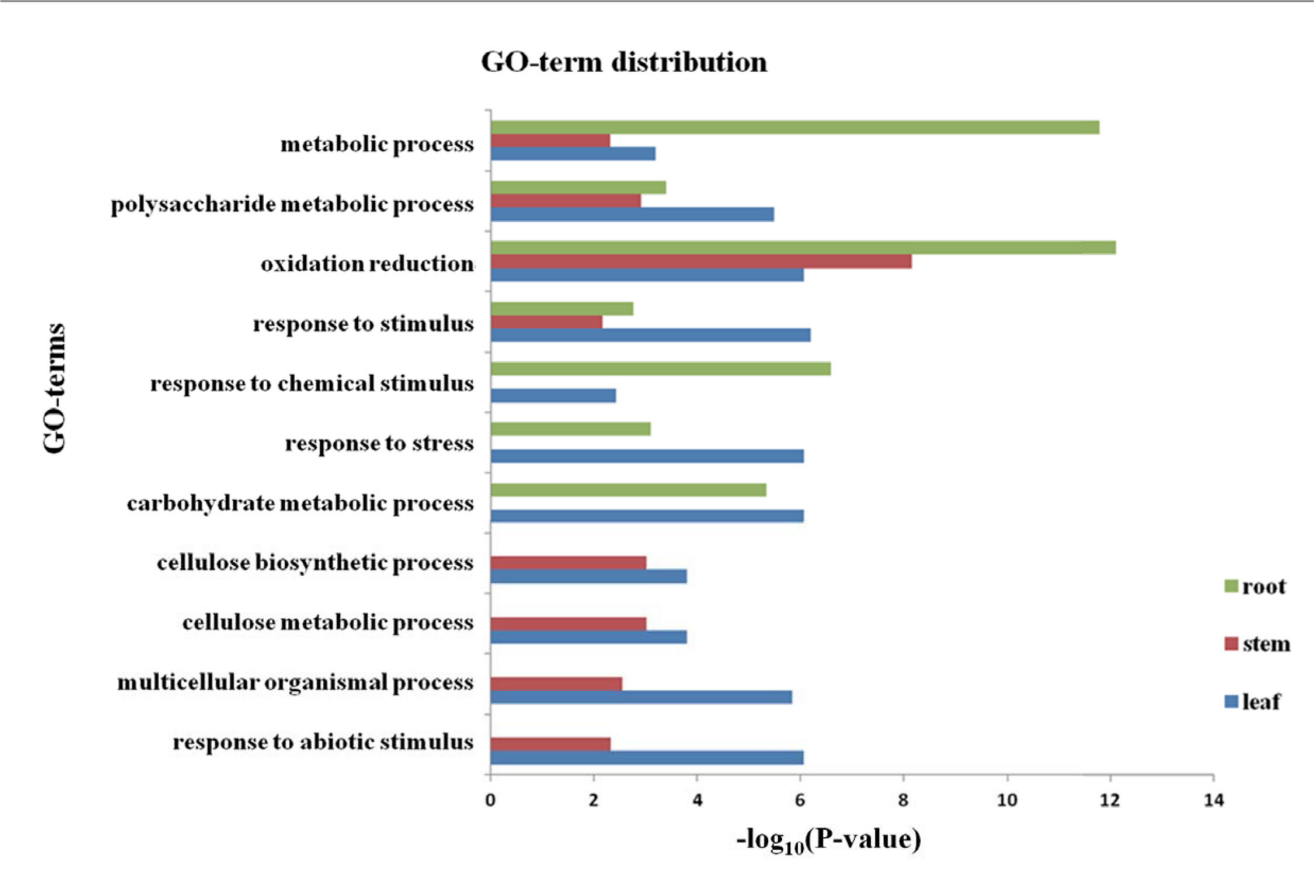
Functional categorization of differentially expressed genes (DEGs) in three tissues. The top four GO terms are functional categorizations (biological process) of the DEGs that were enriched in all three tissues, and the lower seven GO terms are functional categorizations (biological process) of the DEGs enriched in two tissues. The enrichment figure was constructed from significantly enriched GO terms only. The X-axis is the -log_10_ (p-value), which represents the level of enrichment, and the cutoff of the p-value is 0.05. Blue represents leaf tissue, red represents stem tissue, and green represents root tissue.

Then, a GO analysis was conducted with respective DEG terms in the three tissues using the agriGO tool (see methods). Four GO classifications were commonly enriched after comparison among the three tissues, including response to stimulus, oxidation-reduction, polysaccharide metabolic process and metabolic process. The numbers of genes enriched in the three tissues for each commonly enriched GO classification were similar, and only a fraction of these overlapped among the three tissues. For example, there were 190, 167, and 167 genes enriched in leaf, stem and root, respectively, in the “response to stimulus” classification, but only 8 genes were the same, which accounted for approximately 5% of the number in each gene set. Correspondingly, there were 5.8%, 6.3%, and 4.4% commonly enriched genes in leaf, stem and root for oxidation-reduction, polysaccharide metabolic process and metabolic process, respectively. Seven GO classifications were enriched in two tissues. For example, response to chemical stimulus, response to stress and carbohydrate metabolic process were enriched in leaf and root; and cellulose biosynthetic process, cellulose metabolic process, multicellular organismal process and response to abiotic stimulus (which contains drought stress) were enriched in leaf and stem (Fig 3). Furthermore, 74 GO classifications were tissue specific, especially metabolism-related classifications (Fig B in S5 File). Our results suggested that different tissues of plants may have similar stress signal reception and conduction pathways, yet the tissue-specific genes had a greater impact on plant growth and development.

### MAPKKK genes revealed apparently different expression profiles in different tissues

**Fig 4:**
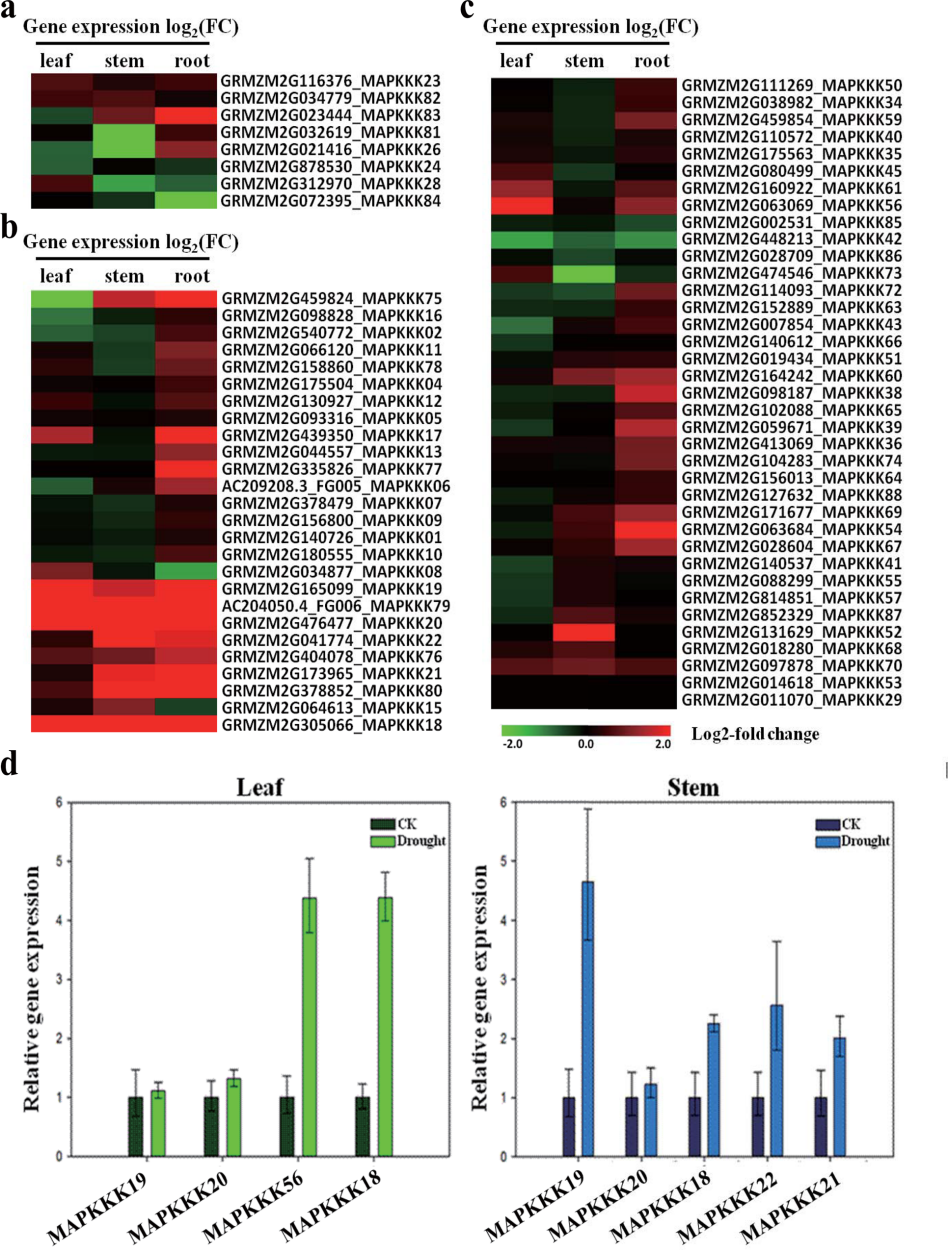
Differential expression of MAPKKKs identified by RNA-seq and qRT-PCR. (a, b and c). Expression patterns of 71 MAPKKK genes in the three tissues. (a). ZIK family genes. (b). MEKK family genes. (c). Raf family genes. (d). Relative expression levels of the MAPKKK genes in various tissues based on qRT-PCR analysis. The expression in leaf is shown on the left. The expression in stem is shown on the right.

Among all the MAPKKK genes, four genes (*ZmMAPKKK18*, *ZmMAPKKK19*, *ZmMAPKKK20* and *ZmMAPKKK56*) in leaf, three genes (*ZmMAPKKK19*, *ZmMAPKKK20* and *ZmMAPKKK21*) in root, and seven genes (*ZmMAPKKK18*, *ZmMAPKKK19*, *ZmMAPKKK20*, *ZmMAPKKK21*, *ZmMAPKKK22*, *ZmMAPKKK26* and *ZmMAPKKK73*) in stem revealed significantly differential expression in response to drought stress. A comprehensive analysis of these three tissues revealed that eight MAPKKK genes may be involved in the response to drought stress. We conducted a cluster analysis of the three MAPKKK sub-families’ expression profiles, including the ZIK family genes(Fig 4A), the MEKK family genes (Fig 4B), and the Raf family genes(Fig 4C). The data suggested that most of the differentially expressed MAPKKKs belonged to the MEKK family. We detected an important gene, *GRMZM2G165099* (*ZmMAPKKK19*), which was predicted to function similar to NPK1 (*Nicotiana* protein kinase), in the MEKK sub-family. The expression of Nicotiana protein kinase (NPK1) has been shown to enhance drought tolerance in transgenic maize [19, 20]. In our results, *ZmMAPKKK19* showed an up-regulation pattern in all three tissues, especially in root, which is in accordance with the previous research.

According to the subcellular localization results (Table A in S1 File) and the expression patterns shown in Fig 4B, the highly up-regulated MAPKKKs in leaf, *ZmMAPKKK17*, *ZmMAPKKK18*, *ZmMAPKKK19*, *ZmMAPKKK20* and *ZmMAPKKK79*, were all located in the chloroplast, which is similar to the genetic mode of their expression in leaf. The results indicated that MAPKKKs may play an important role in drought-related stress. In addition, the pattern of the functional MAPKKKs displays clearly high expression under drought.

Some of the differentially expressed genes in leaf, root, and stem of ZD619 that were identified using RNA-seq were confirmed by qRT-PCR to validate whether these genes were connected with the drought response (Fig 4D). In leaf, the expression levels of *ZmMAPKKK18* and *ZmMAPKKK56* were markedly up-regulated, with greater than 4-fold increases, whereas the changes in *ZmMAPKKK19* and *ZmMAPKKK20* were not significant and were less than two-fold. The expression of genes in stem showed a slight increase in *ZmMAPKKK20*. The expression levels of *ZmMAPKKK18*, *ZmMAPKKK19*, *ZmMAPKKK21* and *ZmMAPKKK22* were up-regulated in stem.

### DEGs were enriched in different pathways in response to drought stress

**Fig 5:**
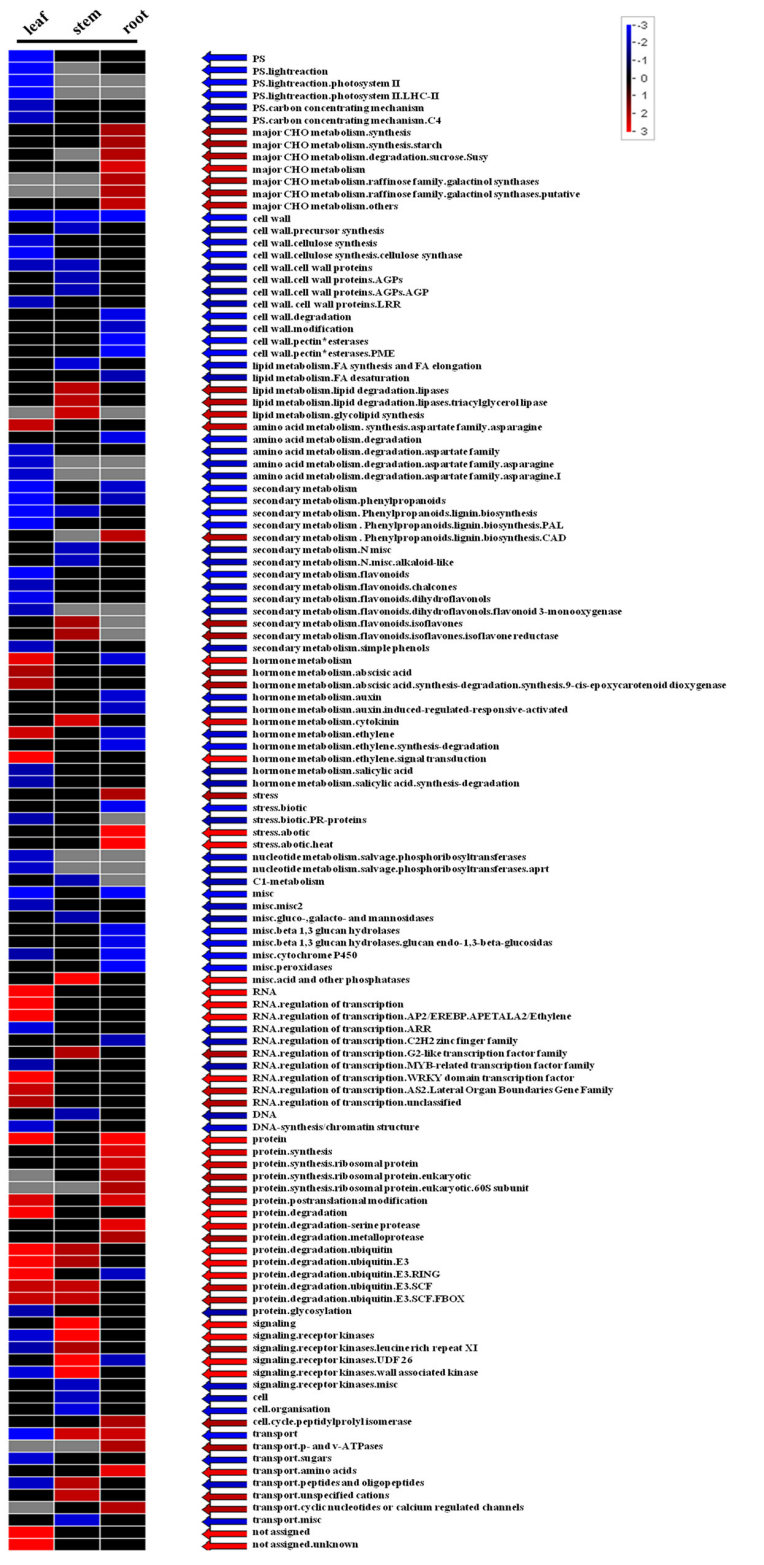
Pathway enrichment of differentially expressed genes involved in different regulatory processes under drought stress. A q-value cutoff of 0.05 was used to select enriched gene sets in all three tissues. The heat map represents the Z-scores obtained from a parametric analysis of gene set enrichment q-values for term enrichment. Red represents enriched genes in the treatment group that were over-represented compared with the control set. Blue represents the enriched genes in the treatment group that were under-represented compared with the control set. The absolute values represent the enrichment level. The bar represents the Z-score region from -3 to 3.

As the above results showed, different tissues showed different responses to drought stress, and the DEGs in the three tissues represented distinct functional categories. Several MAPKKKs were including among the DEGs affected by drought. We investigated how drought regulated the differentially expressed genes, particularly the MAPKKKs, using PageMan to categorize the drought-mediated gene expression data for leaf, stem and root tissues into known metabolic pathways and different regulatory processes (Fig 5).

There were many DEGs enriched in hormone signaling, photosynthesis, CHO metabolism, secondary metabolic processes and regulation of transcription (Fig 5). Genes enriched in hormone signaling included more up-regulated genes in leaf and more down-regulated genes in root. Genes enriched in photosynthesis included many down-regulated genes in leaf. Genes enriched in CHO metabolism included many up-regulated genes in root. Genes enriched in secondary metabolic processes included more down-regulated genes in the three tissues. Genes enriched in regulation of transcription included more up-regulated genes in leaf and stem. To identify important enriched pathways in our DEGs, we analyzed an overview of enrichment in the metabolic processes TCA, starch synthesis and decomposition; proline metabolic pathways (Fig D in S5 File); and hormone signaling pathways (Fig E in S5 File). Genes that were significantly involved in metabolism were more down-regulated in leaf but more up-regulated in root. Genes that were significantly involved in regulation were more down-regulated in stem but up-regulated in leaf and root. These results suggested that the differential regulation of genes in different tissues may lead to particular patterns of regulation.

Furthermore, the overview of regulation revealed three regulated MAPKKKs (*ZmMAPKKK19*, *ZmMAPKKK20* and *ZmMAPKKK56*) in leaf, four regulated MAPKKKs (*ZmMAPKKK20*, *ZmMAPKKK21*, *ZmMAPKKK22* and *ZmMAPKKK26*) in stem, and four regulated MAPKKKs (*ZmMAPKKK17*, *ZmMAPKKK19*, *ZmMAPKKK20* and *ZmMAPKKK21*)in root. *ZmMAPKKK19* and *ZmMAPKKK20* were common in at least two tissues. They exhibited distinctly differential expression after drought stress (Fig 4B). According to the phylogenetic tree (Fig 1B), *ZmMAPKKK17*, *ZmMAPKKK19*, *ZmMAPKKK20*, *ZmMAPKKK21* and *ZmMAPKKK22* belong to the MEKK subfamily. These results indicated that MAPKKKs may have important regulatory functions in drought tolerance in maize.

### Co-expression analysis of DEGs and MAPKKKs

To examine patterns of correlation between the differentially expressed genes and MAPKKKs, we investigated their co-expression using WGCNA [64–66]. All 26,901 filtered transcripts were clustered using WGCNA to obtain an overview of their expression relationships. Networks and models for our data sets were constructed to determine the co-expression value between each pair of genes from our filtered data sets.

**Fig 6:**
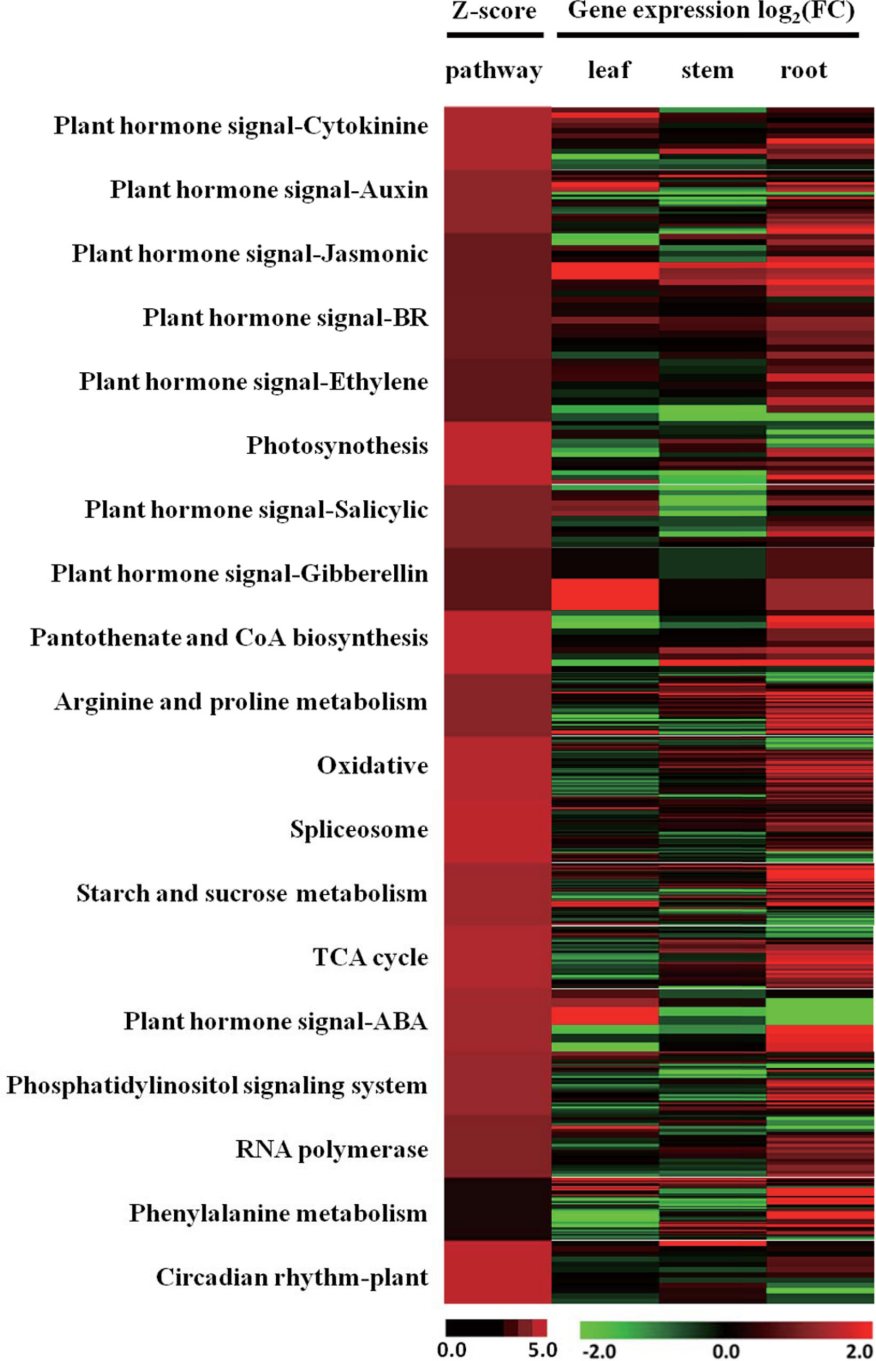
Co-expression analysis of tissue-specific DEGs and MAPKKK genes. The weight value obtained from the WGCNA package was used as a parameter for the parametric analysis of gene co-expression levels. A cutoff of 0.2 was used to select highly co-expressed genes in all three tissues. The left side of the heat map represents the Z-scores obtained from a parametric analysis of gene co-expression. The lower left bar represents the degrees of the Z-score scale. The right side of the heat map represents the expression patterns of the DEGs co-expressed with MAPKKKs that were enriched in the corresponding pathways on the left. The lower right bar represents the log2 of the drought/control ratio.

We focused on the MAPKKKs that were especially co-expressed with DEGs that were enriched in important pathways. After filtering with at least 10 genes, the most significant pathways could be identified, and most of these pathways coexisted in all three tissues. Then, we chose fourteen common and five tissue-specific pathways for further analysis. The heatmap in the left of Fig 6 shows the different co-expression patterns in the three tissues based on their Z-scores. The plant hormone signaling pathway categories oxidation stress, photosynthesis, starch and sucrose metabolism, arginine and proline metabolism, TCA cycle, auxin, cytokinin, gibberellin, ABA, ethylene, BR, jasmonic and salicylic acid presented high Z-scores in the co-expression analysis.

To better understand the relationships between the DEGs and MAPKKKs in the selected pathways, a heat map was constructed to study the expression patterns of single DEGs that were co-expressed with MAPKKKs corresponding to the nineteen enriched pathways (right in Fig 6). The data revealed that the DEGs were highly related to the MAPKKKs (Table D in S1 File). The DEGs that were co-expressed with MAPKKKs in these pathways also showed a broad differential expression pattern. It was found that most of the DEGs were up-regulated in root. In the high Z-score pathways, the photosynthesis pathway contained most of the down-regulated pathway-enriched DEGs, whereas the proline, ethylene and salicylic acids pathways contained many up-regulated pathway-enriched DEGs, especially in root.

Furthermore, thirteen co-expressed MAPKKKs were co-expressed with DEGs in the above nineteen enriched pathways; these MAPKKKs were *ZmMAPKKK11*, *ZmMAPKKK12*, *ZmMAPKKK13*, *ZmMAPKKK22*, *ZmMAPKKK34*, *ZmMAPKKK38*, *ZmMAPKKK39*, *ZmMAPKKK51*, *ZmMAPKKK56*, *ZmMAPKKK65*, *ZmMAPKKK72*, *ZmMAPKKK74* and *ZmMAPKKK83* (Table E in S1 File). Although some MAPKKKs were not found to be co-expressed with DEGs in any of the nineteen pathways, they may play roles in minor pathways under drought stress; these genes included *ZmMAPKKK19* and *ZmMAPKKK21*, among other. *ZmMAPKKK26* and *ZmMAPKKK73* play roles in several pathways. Our results confirmed that *ZmMAPKKK19*, *ZmMAPKKK21*, *ZmMAPKKK22* and *ZmMAPKKK56* were up-regulated in different tissues under drought conditions. *ZmMAPKKK56* in leaf and *ZmMAPKKK22* in stem revealed significantly differential expression in response to drought stress. *ZmMAPKKK21* in stem and root, *ZmMAPKKK19* in all three tissues showed significantly differential expression under drought conditions. These MAPKKKs may be key regulators of the tolerance of plants to drought. The co-expressed DEGs in these nineteen pathways were very peculiar. Only three DEGs were co-expressed with MAPKKKs both in the oxidative and photosynthesis pathways, only five DEGs were co-expressed with MAPKKKs in both the oxidative and TCA pathways. The oxidative pathway, especially ROS, may be the most core element in drought stress. All the results revealed that MAPKKKs provide a link between gene response expression and drought tolerance. The drought stress-responsive MAPKKK genes may be crucial to maize under drought conditions.

### Differential expression levels of the eight MAPKKK genes in response to drought stress in maize

To understand the differences of eight MAPKKK genes in response to drought stress among various maize varieties, a hybrid ZD619 and eight inbred lines of maize were used to further qRT-PCR analysis and biomass determination (Table 2). Plant biomass and kernel yield are usually used as integrative indicators to judge the drought-tolerant potential of plants[67]. In this study, seedling biomass was regarded as a criterion to identify drought tolerance capability. The maize seedling biomass was measured as fresh weight and dry weight and subsequently calculated as drought tolerance index (DTI) to compare the drought response performance of various inbred lines. Results indicated that drought stress exhibited enough influence to elicit differential phenotypes. Each inbred line showed different drought resistance capability, among them X178 was the most tolerant one to drought stress and E28 was the most sensitive one to drought stress.

**Table 2:** The correlation between relative expression levels of the 8 MAPKKK genes based on qRT-PCR analysis and relative fresh and dry biomass in 8 maize inbred lines under well-watered and drought-stress conditions.

	Relative expression in drought based on qRT-PCR								the ratios between drought and control	
	MAPKKK18	MAPKKK19	MAPKKK20	MAPKKK21	MAPKKK22	MAPKKK26	MAPKKK56	MAPKKK73	fresh biomass	Dry biomass
X178	58.4852	20.2990	14.7571	4.2086	7.3785	1.3883	11.7127	1.7777	0.739	0.812
J24	7.9815	4.9933	1.4175	1.5333	1.3272	4.4280	1.6818	7.3107	0.616	0.698
Q319	3.4983	4.3873	5.5854	1.8921	1.8404	1.4777	4.5525	3.8194	0.606	0.695
J853	23.5339	11.6318	5.3889	25.2813	32.7480	1.1783	7.6387	1.4777	0.566	0.634
C8605	4.5106	5.4768	5.1337	1.0930	0.8409	0.7287	4.2772	1.0892	0.503	0.576
B73	1.8877	3.6385	2.4172	0.4687	0.3209	0.9065	1.7053	1.2805	0.491	0.569
200B	8.8356	13.4543	7.4988	1.6021	1.0570	0.4043	1.4880	1.4948	0.483	0.554
E28	2.8219	0.0914	0.2038	2.6208	0.6402	0.8141	1.6663	0.5188	0.421	0.503
Pearson Correlation for fresh biomass	0.78*	0.66	0.71	0.13	0.23	0.45	0.77*	0.45	-	-
Pearson Correlation for dry biomass	0.75*	0.61	0.67	0.09	0.19	0.48	0.74*	0.48	-	-

* indicate significant correlation at P<0.05 respectively.

**Fig 7:**
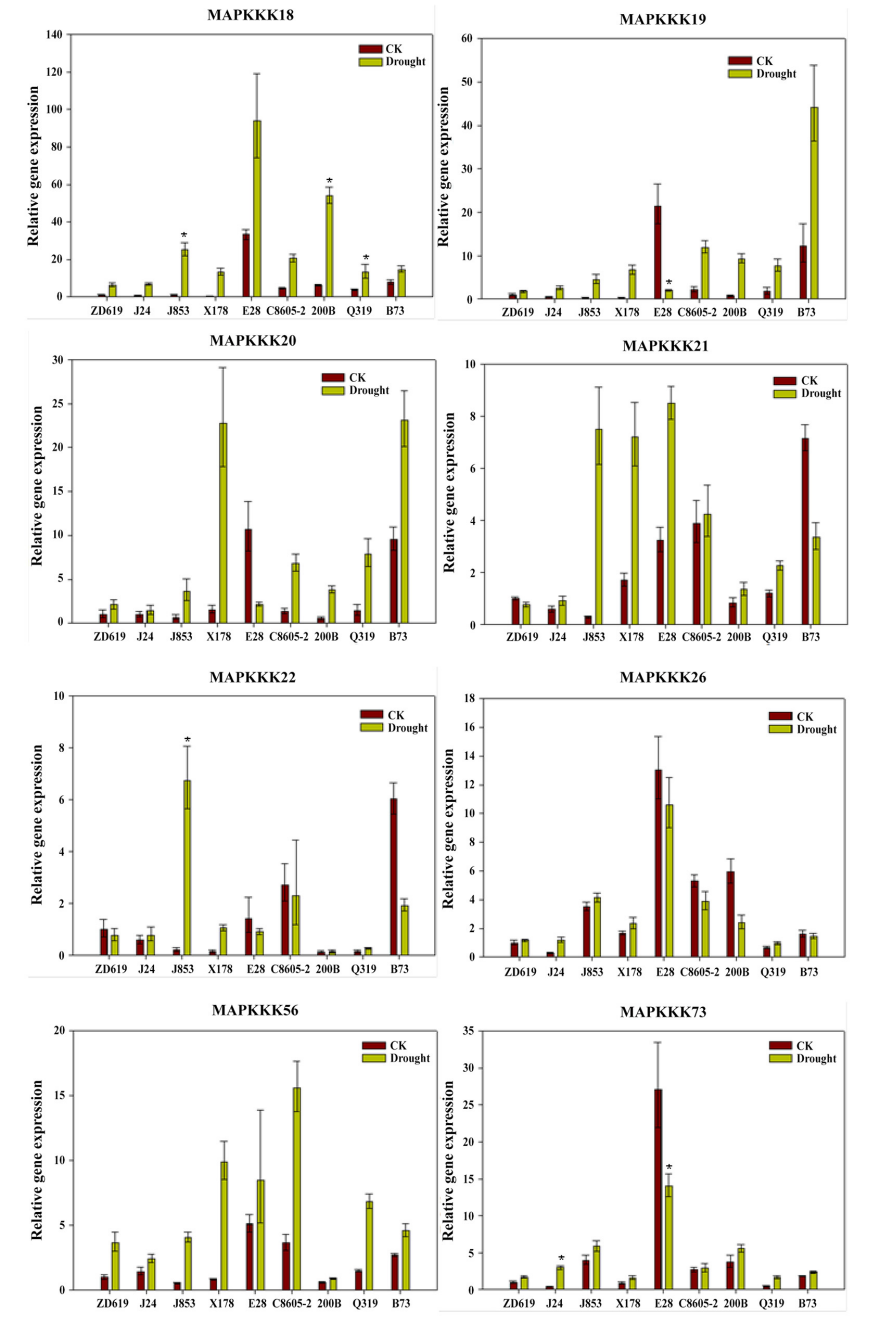
Relative gene expression of the 8 MAPKKK genes in various inbred lines based on qRT-PCR analysis. To determine whether the relative expression levels of the drought stress-responsive MAPKKK genes differed among varieties induced by drought, ZD619 and the 6 inbred maize lines J24, J853, X178, E28, C8605-2, 200B, Q319 and B73 were used. Lines X178, J24 are drought-resistant lines; 200B and E28 have poor drought tolerance. The eight MAPKKK genes are *GRMZM2G305066* (*ZmMAPKKK18*), *GRMZM2G165099* (*ZmMAPKKK19*), *GRMZM2G476477* (*ZmMAPKKK20*), *GRMZM2G173965* (*ZmMAPKKK21*), *GRMZM2G041774* (*ZmMAPKKK22*), *GRMZM2G021416* (*ZmMAPKKK26*), *GRMZM2G063069* (*ZmMAPKKK56*), *GRMZM2G474546* (*ZmMAPKKK73*). * indicates significant differences in comparison with the control at P < 0.05 respectively. Error bars indicate standard deviation for three replicates.

Eight MAPKKK genes for expression analysis by qRT-PCR were shown in Fig 7. The expression levels of the eight genes differed among the nine varieties. The expression levels of *ZmMAPKKK18* and *ZmMAPKKK56* were up-regulated in response to drought stress in all varieties. The expression levels of *ZmMAPKKK19*, *ZmMAPKKK20* and *ZmMAPKKK73* were up-regulated under drought conditions in seven inbred lines except E28. In addition, *ZmMAPKKK21* was up-regulated in seven inbred lines except B73. The expression levels in various inbred lines had significantly different. The inbred line X178, in expression level increased by 58-fold in *ZmMAPKKK18*, 20-fold in *ZmMAPKKK19*, 11-fold in *ZmMAPKKK56*, 8-fold in *ZmMAPKKK20*, 7-fold in *ZmMAPKKK22*, and 4-fold in *ZmMAPKKK21*. In the inbred lines E28 and B73, the expression levels were not significantly changed, and some genes were even down-regulated. In E28, the expression levels of *ZmMAPKKK19*, *ZmMAPKKK20* and *ZmMAPKKK73* were markedly down-regulated. The results indicated that different expression levels of differential expression MAPKKK genes had certain relation with the variety’s drought resistance ability.

We then performed a correlation analysis of the different expression levels of eight MAPKKK genes in eight inbred lines. Pearson’s correlation coefficients were calculated between each gene’s expression pattern and the plant biomass DTI values in the eight inbred maize lines (Table 2). The results suggested that the expression levels of the genes *GRMZM2G305066* (*ZmMAPKKK18*) and *GRMZM2G063069* (*ZmMAPKKK56*) were significantly correlated with changes in plant biomass in response to drought (*P*<0.05). The expression levels of *GRMZM2G165099* (*ZmMAPKKK19*) and *GRMZM2G476477* (*ZmMAPKKK20*) were slightly correlated with plant biomass (P<0.1). This analysis suggested that the eight MAPKKK genes, especially *ZmMAPKKK18* and *ZmMAPKKK56*, were obviously related to biological characteristics of maize under drought stress. Among the eight genes, according to this analysis, *ZmMAPKKK18* and *ZmMAPKKK56* may have crucial functions in the response of maize to drought stress.

## Discussion

### MAPKKK genes are related to drought stress in maize

Plants have developed complicated signaling pathways to adapt to environmental stress. In some species, it has been verified that MAPKKK can be rapidly activated during suffering to drought stress. In Arabidopsis, the mRNA levels of AtMEKK1, which is structurally related to MAPKKK, and AtMPK3, which is structurally related to MAPK, has increased in response to dehydration stress [9]. In rice, a Raf-like MAPKKK gene, *DSM1*, can respond to drought stress through ROS scavenging [51]. The NPK1 (MAPKKK) isolated from tobacco is conserved among different organisms [18]. Transgenic rice and maize with *NPK1* showed significantly improved tolerance to drought [19–21].

In this study, seventy-one MAPKKK genes have been obtained by a computational analysis of the entire maize genome and eight MAPKKK genes that exhibited significantly differential expression in response to drought stress are identified by an RNA-seq analysis. Among these genes, *ZmMAPKKK26* and *ZmMAPKKK73*, which show down-regulated expression in stem under drought stress, are located in the nucleus. *ZmMAPKKK18*, *ZmMAPKKK19*, *ZmMAPKKK20* and *ZmMAPKKK22*, which are up-regulated under drought stress, are located in the chloroplast. Four MAPKKKs are predicted with a serine/threonine-protein kinase function. Notably, *ZmMAPKKK19* is predicted to have NPK1-related protein kinase function. In addition, *ZmMAPKKK19* is highly homologous with *ZmMAPKKK18*. Indeed, the expression levels of these two genes are up-regulated after drought treatment. The results indicate that *ZmMAPKKK18* and *ZmMAPKKK19* may be involved in the drought response. These proteins have high sequence similarity, which may means the correlation with similar expression patterns.

Furthermore, qRT-PCR is performed with eight MAPKKK genes to compare their expression levels in different varieties between drought conditions and well-watered conditions. The results show that the expression levels of these eight MAPKKKs in the nine maize varieties are different. In all the varieties, the relative expression levels of *ZmMAPKKK18* are markedly up-regulated when maize seedlings are under drought stress condition. Additionally, the relative expression levels of *ZmMAPKKK19*, which is predicted to have *NPK1*-related protein kinase function, are up-regulated in seven inbred lines. The genes *ZmMAPKKK18* and *ZmMAPKKK19* may play a crucial role in response to drought stress. However, to elucidate the roles of *ZmMAPKKK18* and *ZmMAPKKK19* in drought tolerance, many details need to be clarified by further research. A comparison of these genes’ expression among the different maize lines show that the abilities of genes to tolerate drought stress probably differ among the different lines.

In our study, the analysis of drought tolerance capability which identified by DTI values indicates that drought stress exhibit enormous influence for the different phenotypes. Among the eight inbred lines, X178 was the most tolerant line to drought stress and E28 was the most sensitive line to drought stress. The correlation analysis reveals that the eight MAPKKK genes, especially *ZmMAPKKK18* and *ZmMAPKKK56*, are significantly correlated with the biological characteristics under drought stress in maize. The data suggests that, among the eight genes, *ZmMAPKKK18* and *ZmMAPKKK56* may have crucial functions in the response of drought stress in maize.

In maize, the expression levels of eight MAPKKK genes are regulated under drought stress, and the results are confirmed in different maize varieties. These observations suggests that the MAPKKK genes may be involved in the drought stress response in maize.

### MAPKKK genes are involved in multiple pathways under drought stress

**Fig 8:**
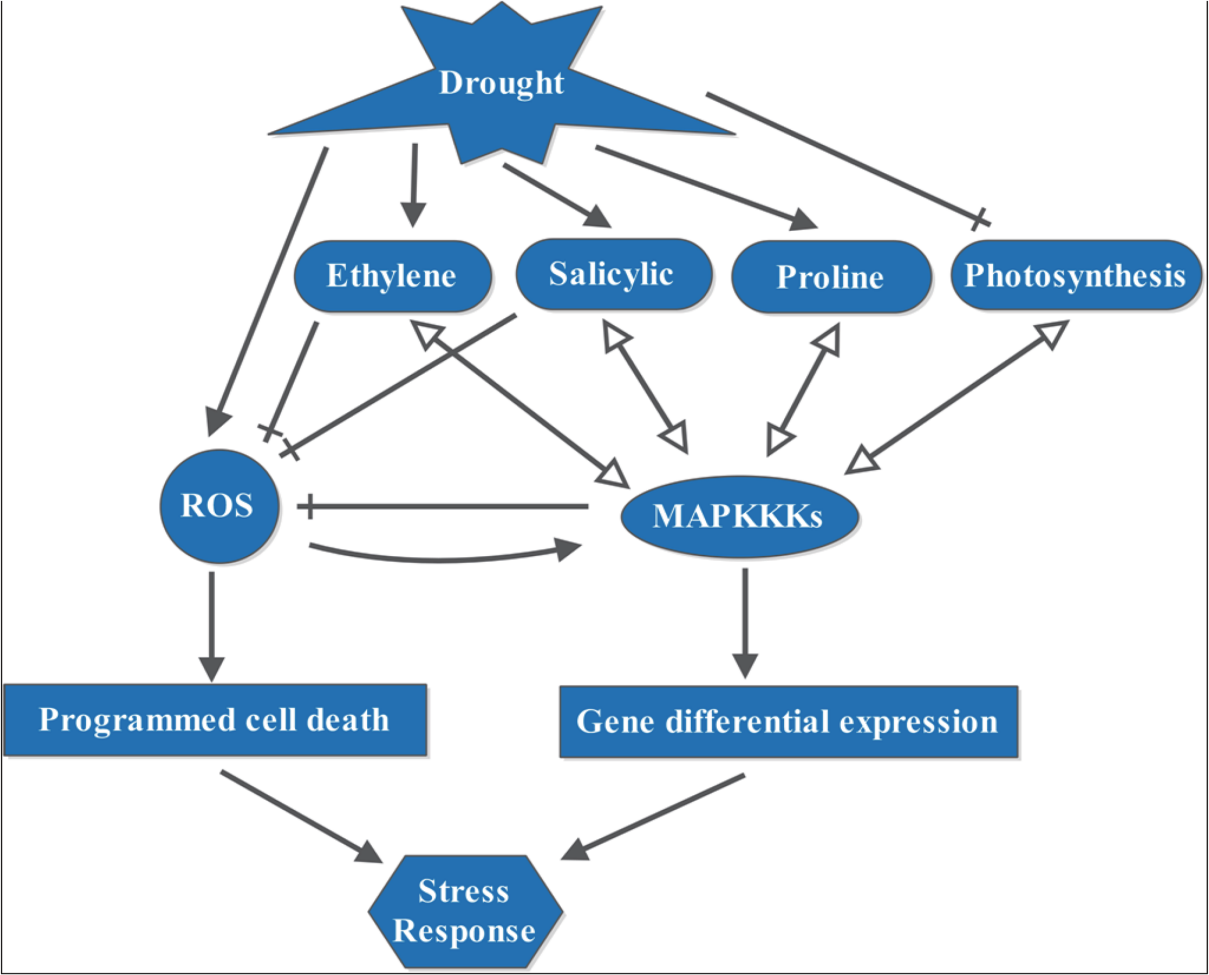
A model of drought stress effects on maize. The colored shapes represent groups of glyphs with similar processes or events. The arrows ending in a solid triangle indicate positive effects. The arrows ending in a transverse line indicate a clear negative influence. The arrows ending in a hollow triangle indicate co-expression. The curved arrows indicate positive feedback.

Plants tend to adapt to drought stress by serial changes. The mechanisms of drought tolerance in plants are complex. The data in our study suggests that drought stress can lead to expression changes in some genes, including MAPKKKs. Under drought stress, several MAPKKKs, especially those that co-expressed with DEGs, are enriched in some important pathways, such as the photosynthesis pathway, hormone signal transduction pathway, oxidation pathway, and protein modifications(including proline) pathway (Fig 8). In the photosynthesis pathway, there are more down-regulated DEGs in leaf. There are many up-regulated DEGs in the three tissues, especially in root. There are obviously more up-regulated DEGs in oxidation and protein modification (including proline) pathways, primarily in root. It is well known that drought stress encumbers photosynthetic carbon fixation by limiting the entry of CO_2_ into leaves via down-regulating the photosynthetic metabolism or accelerating stomatal closure. Furthermore, proline synthesis moderately increases under drought stress, which can help plants to resist drought stress. The accumulation of proline in transgenic plants is associated with higher biomass under drought stress [5, 6]. In our study, the enriched DEGs are down-regulated in the photosynthesis pathway in leaf and are up-regulated in proline modification, which are in accordance with previous studies. Some MAPKKKs are found to be co-expressed with DEGs in these pathways, which suggests that MAPKKKs are involved in the photosynthesis pathway and proline modification pathway under drought stress condition. Among them, the down-regulated *ZmMAPKKK26* and *ZmMAPKKK73* are co-expressed with DEGs in the photosynthesis pathway, while the up-regulated *ZmMAPKKK19*, *ZmMAPKKK21*, *ZmMAPKKK22* and *ZmMAPKKK56* are co-expressed with DEGs in proline modification pathway.

Moreover, the accumulation of ethylene and salicylic acid may be caused by water stress. Several earlier investigations have found that some ethylene response factor genes and endogenous salicylic acid levels can induce water stress tolerance in plants [68–71]. Seed which is pretreated with SA promotes plant vigor index under drought stress condition [60]. A study in *Medicago* and Arabidopsis plants showed that a MAPK pathway was involved in ethylene signaling [61]. Drought inhibits maize growing by causing oxidative damage to biological membranes and disturbing the water status in tissues. Reactive oxygen species (ROS) are central signaling molecules in response to drought. To reduce the toxicity of ROS, plant cells have developed an antioxidative system. The increasing ROS to toxic levels can activate PCD to remove damaged cells. Ethylene and salicylic acid response factor proteins may be connected to the ROS pathway through defending cells against ROS attacks. Salicylic acid can enhance the antioxidant status and the tolerance to drought stress [62]. Ethylene plays an important role in lowering ROS generation and protecting plants under drought stress [63]. Ethylene can modulate the programmed cell death induced by O_3_ exposure [72]. Many studies have reported that MAPK cascades play crucial roles in ROS signaling pathway under water stress. MAPK, which is induced by oxidative stress, can promote ROS scavenging. Arabidopsis *MKK4* increases plant drought tolerance and decreases the production of ROS [73]. Tolerance to drought stress, which is enhanced by *NPK1*, may activate the oxidative signaling pathway [19]. In tobacco, *GbMPK3* increases ROS scavenging under drought stress [74]. The MAPKKKs that are activated under drought stress may promote ROS scavenging to control the concentrations of ROS. In our study, DEGs are enriched in the ethylene and SA hormone signal transduction pathways. The expression levels of most of the genes related to salicylic acid and ethylene are up-regulated. The up-regulation of proteins may be involved in redox homeostasis, ROS scavenging and its damaging effects on proteins and DNA. In addition, some MAPKKKs are co-expressed with DEGs in ethylene and SA plant hormone signal transduction pathways, such as *ZmMAPKKK19*, *ZmMAPKKK21*, *ZmMAPKKK22* and *ZmMAPKKK56*. The data supports the theory that MAPKKKs may promote ROS scavenging by the ethylene and SA pathways in response to drought. The ethylene and SA hormone signal transduction pathway-related genes, especially in root, show an up-regulation pattern, which reveals that roots may play a key role in response to drought stress. Under drought condition, MAPKKKs are activated by ROS, which leads to the differential expression of some genes. The expression levels of eight MAPKKK genes are increased under drought treatment. The genes *ZmMAPKKK18* and *ZmMAPKKK19*, which are highly homologous to NPK1, are up-regulated in leaf, stem and root, which may play roles in regulating the response to drought stress. The differentially expressed MAPKKKs, *ZmMAPKKK19*, *ZmMAPKKK21*, *ZmMAPKKK22* and *ZmMAPKKK56* are co-expressed with some DEGs in most pathways.

In conclusion, seventy-one MAPKKK genes in maize are identified in our study. Among them, 8 differentially expressed MAPKKK genes responsive to drought stress are obtained. The results suggest that MAPKKKs may react to drought stress through various pathways. To our knowledge, this is the first study of the expression of maize MAPKKK genes in relation to drought stress. The results of our study should provide further information about the characteristics of MAPKKKs in maize and their roles in the response to drought stress. Further biological functional analyses of MAPKKKs and their downstream MAPKK and MAPK targets will help elucidate the mechanisms underlying the response of MAPK cascades to drought in maize.

## Supporting Information

S1 File Supplementary tables. S1 File contains five Supplementary tables: Table A. 71 predictive MAPKKKs in maize. Table B. The quality of the RNA-seq data for Q20 and Q30. Table C. RNA-seq data and mapping rates. Table D. The co-expressed MAPKKKs with DEGs in the enriched pathway. Table E. The common co-expressed MAPKKKs with our DEGs in the enriched pathway.
(DOC)

S2 File Differently Expressed Genes Responded to drought in leaf, stem and root. (XLSX)

S3 File Genes with different regulated pattern in three organs. (XLSX)

S4 File Primers for qPCR. (XLSX)

S5 File Contains five supplementary figures: Fig A. Conserved domains of MAPKKKs in three sub-families. (a).The conserved motif GTPEFMAPE (L/V) (Y/F) in the ZIK family. (b).The conserved motif G (T/S) Px (W/F) MAPEV in the MEKK family. (c).The conserved motif GTxx (W/Y) MAPE in the Raf family. Fig B. Functional categorization (biological process) of DEGs that are enriched in only one tissue. Blue represents leaf. Red represents stem. Green represents root. The enrichment figure was constructed of significantly enriched GO terms only. The X-axis is the -log_10_ (p-value), which represents the level of enrichment, and the p-value cutoff was 0.05. Fig C. Venn analysis for the DEGs with up-regulated and down-regulated in different organs. Each circle represents an organ with different regulated pattern. Fig D. Differentially expressed transcripts involved in different metabolic processes under drought stress. (a, b and c) represent drought-mediated expression changes in different metabolic processes in the leaf, stem and root meristem, respectively. The images were obtained using MapMan and show the different functional categories that fulfilled the criteria for differential expression (a p-value less than 0.05 and greater than 2-fold change). Fig E. Differentially expressed transcripts involved in different regulatory processes under drought stress. (a, b and c) represent drought-mediated expression changes in different regulatory processes in the leaf, stem and root meristem, respectively. The images were obtained using MapMan and show the different functional categories that fulfilled the criteria for differential expression (a p-value less than 0.05 and greater than 2-fold change). (ZIP)

## References

[1] Chaves MM, Flexas J, Pinheiro C. Photosynthesis under drought and salt stress: regulation mechanisms from whole plant to cell. Annals of Botany. 2009;103(4):551–60. 10.1093/aob/mcn125 . 18662937 PMC2707345

[2] Krugman T, Peleg Z, Quansah L, Chague V, Korol AB, Nevo E, et al Alteration in expression of hormone-related genes in wild emmer wheat roots associated with drought adaptation mechanisms. Functional & Integrative Genomics. 2011;11(4):565–83. 10.1007/s10142-011-0231-6 . 21656015

[3] Kakumanu A, Ambavaram MMR, Klumas C, Krishnan A, Batlang U, Myers E, et al Effects of Drought on Gene Expression in Maize Reproductive and Leaf Meristem Tissue Revealed by RNA-Seq. Plant Physiology. 2012;160(2):846–67. 10.1104/pp.112.200444 . 22837360 PMC3461560

[4] Xu J, Yuan Y, Xu Y, Zhang G, Guo X, Wu F, et al Identification of candidate genes for drought tolerance by whole-genome resequencing in maize. Bmc Plant Biology. 2014;14 83 10.1186/1471-2229-14-83 . 24684805 PMC4021222

[5] de Ronde JA, van der Mescht A, Steyn HSF. Proline accumulation in response to drought and heat stress in cotton. African crop science journal. 2000;8(1):85–91.

[6] Roosens NH, Al Bitar F, Loenders K, Angenon G, Jacobs M. Overexpression of ornithine-δ-aminotransferase increases proline biosynthesis and confers osmotolerance in transgenic plants. Molecular Breeding. 2002;9(2):73–80. 10.1023/a:1026791932238 .

[7] Frye CA, Tang D, Innes RW. Negative regulation of defense responses in plants by a conserved MAPKK kinase. Proceedings of the National Academy of Sciences of the United States of America. 2001;98(1):373–8. 11114160 10.1073/pnas.98.1.373 PMC14597

[8] Morris PC. MAP kinase signal transduction pathways in plants. New Phytologist. 2001;151(1):67–89. 10.1046/j.1469-8137.2001.00167.x 33873387

[9] Mizoguchi T, Irie K, Hirayama T, Hayashida N, Yamaguchi-Shinozaki K, Matsumoto K, et al A gene encoding a mitogen-activated protein kinase kinase kinase is induced simultaneously with genes for a mitogen-activated protein kinase and an S6 ribosomal protein kinase by touch, cold, and water stress in *Arabidopsis thaliana* . Plant Biology. 1996;93:765–9. 10.1073/pnas.93.2.765 PMC40129 8570631

[10] Agrawal GK, Rakwal R, Iwahashi H. Isolation of novel rice (*Oryza sativa* L.) multiple stress responsive MAP kinase gene, *OsMSRMK2*, whose mRNA accumulates rapidly in response to environmental cues. Biochemical and Biophysical Research Communications. 2002;294(5):1009–16. 10.1016/S0006-291X(02)00571-5. 12074577

[11] Jeong MJ, Lee SK, Kim BG, Kwon TR, Cho WS, Park YT, et al A rice (*Oryza sativa* L.) MAP kinase gene, *OsMAPK44*, is involved in response to abiotic stresses. Plant Cell Tissue and Organ Culture. 2006;85(2):151–60. 10.1007/s11240-005-9064-0 .

[12] Xiong LZ, Yang YN. Disease resistance and abiotic stress tolerance in rice are inversely modulated by an abscisic acid-inducible mitogen-activated protein kinase. Plant Cell. 2003;15(3):745–59. 10.1105/tpc.008714 . 12615946 PMC150027

[13] Kumar K, Rao KP, Sharma P, Sinha AK. Differential regulation of rice mitogen activated protein kinase kinase (MKK) by abiotic stress. Plant Physiology and Biochemistry. 2008;46(10):891–7. 10.1016/j.plaphy.2008.05.014. 10.1016/j.plaphy.2008.05.014 18619847

[14] Wang J, Ding H, Zhang A, Ma F, Cao J, Jiang M. A Novel Mitogen-Activated Protein Kinase Gene in Maize (*Zea mays*), *ZmMPK3*, is Involved in Response to Diverse Environmental Cues. Journal of Integrative Plant Biology. 2010;52(5):442–52. 10.1111/j.1744-7909.2010.00906.x . 20537040

[15] Jonak C, Kiegerl S, Ligterink W, Barker PJ, Huskisson NS, Hirt H. Stress signaling in plants: a mitogen-activated protein kinase pathway is activated by cold and drought. Proceedings of the National Academy of Sciences of the United States of America. 1996;93(20):11274–9. 8855346 10.1073/pnas.93.20.11274 PMC38320

[16] Virk N, Liu B, Zhang H, Li X, Zhang Y, Li D, et al Tomato SlMPK4 is required for resistance against Botrytis cinerea and tolerance to drought stress. Acta Physiologiae Plantarum. 2013;35(4):1211–21. 10.1007/s11738-012-1160-2 .

[17] Ichimura K, Mizoguchi T, Yoshida R, Yuasa T, Shinozaki K. Various abiotic stresses rapidly activate *Arabidopsis* MAP kinases ATMPK4 and ATMPK6. The Plant Journal. 2000;24(5):655–65. 11123804 10.1046/j.1365-313x.2000.00913.x

[18] Banno H, Hirano K, Nakamura T, Irie K, Nomoto S, Matsumoto K, et al *NPK1*, a tobacco gene that encodes a protein with a domain homologous to yeast BCK1, STE11, and Byr2 protein kinases. Molecular and cellular biology. 1993;13(8):4745–52. 8336712 10.1128/mcb.13.8.4745-4752.1993 PMC360100

[19] Shou HX, Bordallo P, Wang K. Expression of the *Nicotiana* protein kinase (NPK1) enhanced drought tolerance in transgenic maize. Journal of Experimental Botany. 2004;55(399):1013–9. 10.1093/jxb/erb129 . 15073214

[20] Muoma JVO, Ombori O. *Agrobacterium*-Mediated Transformation of Selected Kenyan Maize (*Zea mays* L.) Genotypes by Introgression of Nicotiana Protein Kinase (*npk1*) to Enhance Drought Tolerance. American Journal of Plant Sciences. 2014;5.

[21] Xiao B-Z, Chen X, Xiang C-B, Tang N, Zhang Q-F, Xiong L-Z. Evaluation of Seven Function-Known Candidate Genes for their Effects on Improving Drought Resistance of Transgenic Rice under Field Conditions. Molecular Plant. 2009;2(1):73–83. 10.1093/mp/ssn068 . 19529831 PMC2639742

[22] Widmann C, Gibson S, Jarpe MB, Johnson GL. Mitogen-Activated Protein Kinase: Conservation of a Three-Kinase Module From Yeast to Human. Physiological Society. 1999;79(1):143–80. 10.1152/physrev.1999.79.1.143 9922370

[23] Agrawal GK, Agrawal SK, Shibato J, Iwahashi H, Rakwal R. Novel rice MAP kinases OsMSRMK3 and OsWJUMK1 involved in encountering diverse environmental stresses and developmental regulation. Biochemical and Biophysical Research Communications. 2003;300(3):775–83. 10.1016/S0006-291X(02)02868-1. 12507518

[24] Andreasson E, Ellis B. Convergence and specificity in the *Arabidopsis* MAPK nexus. Trends in Plant Science. 2010;15(2):106–13. 10.1016/j.tplants.2009.12.001. 10.1016/j.tplants.2009.12.001 20047850

[25] Jonak C, Ökrész L, Bögre L, Hirt H. Complexity, Cross Talk and Integration of Plant MAP Kinase Signalling. Current Opinion in Plant Biology. 2002;5(5):415–24. 10.1016/S1369-5266(02)00285-6. 12183180

[26] Lewis TS, Shapiro PS, Ahn NG. Signal Transduction through MAP Kinase Cascades In: George FVW, George K, editors. Advances in Cancer Research. Volume 74: Academic Press; 1998 p. 49–139. 9561267 10.1016/s0065-230x(08)60765-4

[27] Madhani HD, Fink GR. The riddle of MAP kinase signaling specificity. Trends in Genetics. 1998;14(4):151–5. 10.1016/S0168-9525(98)01425-5. 9594663

[28] Teige M, Scheikl E, Eulgem T, Dóczi R, Ichimura K, Shinozaki K, et al The MKK2 Pathway Mediates Cold and Salt Stress Signaling in *Arabidopsis* . Molecular Cell. 2004;15(1):141–52. 10.1016/j.molcel.2004.06.023. 15225555

[29] Tena G, Asai T, Chiu W-L, Sheen J. Plant mitogen-activated protein kinase signaling cascades. Current Opinion in Plant Biology. 2001;4(5):392–400. 10.1016/S1369-5266(00)00191-6. 11597496

[30] Wrzaczek M, Hirt H. Plant MAP kinase pathways: how many and what for? Biology of the Cell. 2001;93(1–2):81–7. 10.1016/S0248-4900(01)01121-2. 11730326

[31] Huang HJ, Fu SF, Tai YH, Chou WC, Huang DD. Expression of *Oryza sativa* MAP kinase gene is developmentally regulated and stress-responsive. Physiologia Plantarum. 2002;114(4):572–80. 10.1034/j.1399-3054.2002.1140410.x . 11975731

[32] Matsuoka D, Nanmori T, Sato K, Fukami Y, Kikkawa U, Yasuda T. Activation of AtMEK1, an *Arabidopsis* mitogen-activated protein kinase kinase, *in vitro* and *in vivo*: analysis of active mutants expressed in *E*. *coli* and generation of the active form in stress response in seedlings. Plant Journal. 2002;29(5):637–47. 10.1046/j.0960-7412.2001.01246.x . 11874576

[33] Schaeffer HJ, Weber MJ. Mitogen-activated protein kinases: specific messages from ubiquitous messengers. Molecular and cellular biology. 1999;19(4):2435–44. 10082509 10.1128/mcb.19.4.2435 PMC84036

[34] Mizoguchi T, Ichimura K, Yoshida R, Shinozaki K. MAP kinase cascades in *Arabidopsis*: their roles in stress and hormone responses. MAP Kinases in Plant Signal Transduction. 2000;27:29–38. 10.1007/978-3-540-49166-8_3 10533196

[35] Group M, Ichimura K, Shinozaki K, Tena G, Sheen J, Henry Y, et al Mitogen-activated protein kinase cascades in plants: a new nomenclature. Trends in Plant Science. 2002;7(7):301–8. 10.1016/S1360-1385(02)02302-6. 12119167

[36] Hamel L-P, Nicole M-C, Sritubtim S, Morency M-J, Ellis M, Ehlting J, et al Ancient signals: comparative genomics of plant MAPK and MAPKK gene families. Trends in Plant Science. 2006;11(4):192–8. 10.1016/j.tplants.2006.02.007. 16537113

[37] Rao KP, Richa T, Kumar K, Raghuram B, Sinha AK. In Silico Analysis Reveals 75 Members of Mitogen-Activated Protein Kinase Kinase Kinase Gene Family in Rice. DNA Research. 2010;17(3):139–53. 10.1093/dnares/dsq011 . 20395279 PMC2885274

[38] Zhang M, Pan J, Kong X, Zhou Y, Liu Y, Sun L, et al *ZmMKK3*, a novel maize group B mitogen-activated protein kinase kinase gene, mediates osmotic stress and ABA signal responses. Journal of Plant Physiology. 2012;169(15):1501–10. 10.1016/j.jplph.2012.06.008. 10.1016/j.jplph.2012.06.008 22835533

[39] Kong X, Pan J, Zhang M, Xing X, Zhou Y, Liu Y, et al *ZmMKK4*, a novel group C mitogen-activated protein kinase kinase in maize (*Zea mays*), confers salt and cold tolerance in transgenic *Arabidopsis* . Plant Cell and Environment. 2011;34(8):1291–303. 10.1111/j.1365-3040.2011.02329.x . 21477122

[40] Wu T, Kong X-P, Zong X-J, Li D-P, Li D-Q. Expression analysis of five maize MAP kinase genes in response to various abiotic stresses and signal molecules. Molecular Biology Reports. 2011;38(6):3967–75. 10.1007/s11033-010-0514-3 . 21120617

[41] Liu Y, Zhou Y, Liu L, Sun L, Zhang M, Liu Y, et al Maize *ZmMEK1* is a single-copy gene. Molecular Biology Reports. 2012;39(3):2957–66. 10.1007/s11033-011-1057-y . 21691709

[42] Pan J, Zhang M, Kong X, Xing X, Liu Y, Zhou Y, et al *ZmMPK17*, a novel maize group D MAP kinase gene, is involved in multiple stress responses. Planta. 2012;235(4):661–76. 10.1007/s00425-011-1510-0 . 22006107

[43] Berberich T, Sano H, Kusano T. Involvement of a MAP kinase, ZmMPK5, in senescence and recovery from low-temperature stress in maize. Molecular and General Genetics MGG. 1999;262(3):534–42. 10589842 10.1007/s004380051115

[44] Liu Y, Zhang D, Wang L, Li D. Genome-Wide Analysis of Mitogen-Activated Protein Kinase Gene Family in Maize. Plant Molecular Biology Reporter. 2013;31(6):1446–60. 10.1007/s11105-013-0623-y .

[45] Kong X, Pan J, Zhang D, Jiang S, Cai G, Wang L, et al Identification of mitogen-activated protein kinase kinase gene family and MKK–MAPK interaction network in maize. Biochemical and Biophysical Research Communications. 2013;441(4):964–9. 10.1016/j.bbrc.2013.11.008. 10.1016/j.bbrc.2013.11.008 24220337

[46] Jouannic S, Hamal A, Leprince AS, Tregear JW, Kreis M, Henry Y. Plant MAP kinase kinase kinases structure, classification and evolution. Gene. 1999;233(1–2):1–11. 10.1016/S0378-1119(99)00152-3. 10375615

[47] Jouannic S, Hamal A, Leprince A-S, Tregear JW, Kreis M, Henry Y. Characterisation of novel plant genes encoding MEKK/STE11 and RAF-related protein kinases. Gene. 1999;229(1–2):171–81. 10.1016/S0378-1119(99)00012-8. 10095117

[48] Ligterink W, Hirt H. Mitogen-activated protein (MAP) kinase pathways in plants: Versatile signaling tools International Review of Cytology. Volume 201: Academic Press; 2001 p. 209–75. 11057833 10.1016/s0074-7696(01)01004-x

[49] Champion A, Picaud A, Henry Y. Reassessing the MAP3K and MAP4K relationships. Trends in Plant Science. 2004;9(3):123–9. 10.1016/j.tplants.2004.01.005. 15003235

[50] Rodriguez MC, Petersen M, Mundy J. Mitogen-activated protein kinase signaling in plants. Annual review of plant biology. 2010;61:621–49. 10.1146/annurev-arplant-042809-112252 20441529

[51] Ning J, Li X, Hicks LM, Xiong L. A Raf-Like MAPKKK Gene *DSM1* Mediates Drought Resistance through Reactive Oxygen Species Scavenging in Rice. Plant Physiology. 2010;152(2):876–90. 10.1104/pp.109.149856 . 20007444 PMC2815886

[52] Kovtun Y, Chiu WL, Zeng W, Sheen J. Suppression of auxin signal transduction by a MAPK cascade in higher plants. Nature. 1998;395(6703):716–20. 9790195 10.1038/27240

[53] Kovtun Y, Chiu WL, Tena G, Sheen J. Functional analysis of oxidative stress-activated mitogen-activated protein kinase cascade in plants. Proceedings of the National Academy of Sciences of the United States of America. 2000;97(6):2940–5. 10717008 10.1073/pnas.97.6.2940 PMC16034

[54] Cai G, Wang G, Wang L, Liu Y, Pan J, Li D. A maize mitogen-activated protein kinase kinase, ZmMKK1, positively regulated the salt and drought tolerance in transgenic *Arabidopsis* . Journal of Plant Physiology. 2014;171(12):1003–16. 10.1016/j.jplph.2014.02.012. 10.1016/j.jplph.2014.02.012 24974327

[55] Lin F, Ding H, Wang J, Zhang H, Zhang A, Zhang Y, et al Positive feedback regulation of maize NADPH oxidase by mitogen-activated protein kinase cascade in abscisic acid signalling. Journal of Experimental Botany. 2009;60(11):3221–38. 10.1093/jxb/erp157 . 19592501 PMC2718220

[56] Kong X, Sun L, Zhou Y, Zhang M, Liu Y, Pan J, et al *ZmMKK4* regulates osmotic stress through reactive oxygen species scavenging in transgenic tobacco. Plant Cell Reports. 2011;30(11):2097–104. 10.1007/s00299-011-1116-9 . 21735232

[57] Kong X, Lv W, Zhang D, Jiang S, Zhang S, Li D. Genome-Wide Identification and Analysis of Expression Profiles of Maize Mitogen-Activated Protein Kinase Kinase Kinase. Plos One. 2013;8(2). e57714 10.1371/journal.pone.0057714 . PMC3584077 23460898

[58] Trapnell C, Roberts A, Goff L, Pertea G, Kim D, Kelley DR, et al Differential gene and transcript expression analysis of RNA-seq experiments with TopHat and Cufflinks. Nature Protocols. 2012;7(3):562–78. 10.1038/nprot.2012.016 . 22383036 PMC3334321

[59] Ding Y, Virlouvet L, Liu N, Riethoven JJ, Fromm M, Avramova Z. Dehydration stress memory genes of Zea mays; comparison with Arabidopsis thaliana. Bmc Plant Biology. 2014;14 141 10.1186/1471-2229-14-141 . 24885787 PMC4081654

[60] Salvo SA, Hirsch CN, Buell CR, Kaeppler SM, Kaeppler HF. Whole Transcriptome Profiling of Maize during Early Somatic Embryogenesis Reveals Altered Expression of Stress Factors and Embryogenesis-Related Genes. Plos One. 2014;9(10). 10.1371/journal.pone.0111407 PMC4214754 25356773

[61] Dolezal AL, Shu X, Obrian GR, Nielsen DM, Woloshuk CP, Boston RS, et al Aspergillus flavus infection induces transcriptional and physical changes in developing maize kernels. Frontiers in Microbiology. 2014;5 384 10.3389/fmicb.2014.00384 . 25132833 PMC4117183

[62] Yue X, Zhao X, Fei Y, Zhang X. Correlation of Aquaporins and Transmembrane Solute Transporters Revealed by Genome-Wide Analysis in Developing Maize Leaf. Comparative and Functional Genomics. 2012 546930 10.1155/2012/546930 . 23055821 PMC3463914

[63] Bosch M, Mayer CD, Cookson A, Donnison IS. Identification of genes involved in cell wall biogenesis in grasses by differential gene expression profiling of elongating and non-elongating maize internodes. Journal of Experimental Botany. 2011;62(10):3545–61. 10.1093/jxb/err045 . 21402660 PMC3130177

[64] Ouaked F, Rozhon W, Lecourieux D, Hirt H. A MAPK pathway mediates ethylene signaling in plants. Embo Journal. 2003;22(6):1282–8. 10.1093/emboj/cdg131 . 12628921 PMC151067

[65] Senaratna T, Touchell D, Bunn E, Dixon K. Acetyl salicylic acid (Aspirin) and salicylic acid induce multiple stress toleranca in bean and tomato plants. Plant Growth Regulation. 2000;30(2):157–61.

[66] Wu L, Zhang Z, Zhang H, Wang X-C, Huang R. Transcriptional Modulation of Ethylene Response Factor Protein JERF3 in the Oxidative Stress Response Enhances Tolerance of Tobacco Seedlings to Salt, Drought, and Freezing. Plant Physiology. 2008;148(4):1953–63. 10.1104/pp.108.126813 . 18945933 PMC2593663

[67] Collins CN, Tardieu F, Tuberosa R. Quantitative trait loci and crop performance under abiotic stress: where do we stand?. Plant Physiology. 2008;147:469–86. 10.1104/pp.108.118117 18524878 PMC2409033

[68] Zhang H, Liu W, Wan L, Li F, Dai L, Li D, et al Functional analyses of ethylene response factor JERF3 with the aim of improving tolerance to drought and osmotic stress in transgenic rice. Transgenic Research. 2010;19(5):809–18. 10.1007/s11248-009-9357-x . 20087656

[69] Zhang Z, Li F, Li D, Zhang H, Huang R. Expression of ethylene response factor JERF1 in rice improves tolerance to drought. Planta. 2010;232(3):765–74. 10.1007/s00425-010-1208-8 . 20574667

[70] Barkosky RR, Einhellig FA. Effects of salicylic acid on plant-water relationships. Journal of Chemical Ecology. 1993;19(2):237–47. 10.1007/BF00993692 24248871

[71] He Q, Zhao S, Ma Q, Zhang Y, Huang L, Li G, et al Endogenous salicylic acid levels and signaling positively regulate *Arabidopsis* response to polyethylene glycol-simulated drought stress. Journal of Plant Growth Regulation. 2014.

[72] Overmyer K, Brosché M, Kangasjärvi J. Reactive oxygen species and hormonal control of cell death. Trends in Plant Science. 2003;8(7):335–42. 10.1016/S1360-1385(03)00135-3. 12878018

[73] Kim S-H, Woo D-H, Kim J-M, Lee S-Y, Chung WS, Moon Y-H. *Arabidopsis* MKK4 mediates osmotic-stress response via its regulation of MPK3 activity. Biochemical and Biophysical Research Communications. 2011;412(1):150–4. 10.1016/j.bbrc.2011.07.064. 10.1016/j.bbrc.2011.07.064 21806969

[74] Long L, Gao W, Xu L, Liu M, Luo X, He X, et al *GbMPK3*, a mitogen-activated protein kinase from cotton, enhances drought and oxidative stress tolerance in tobacco. Plant Cell Tissue and Organ Culture. 2014;116(2):153–62. 10.1007/s11240-013-0392-1 .

