# Supplementary material for: RNA-Seq Analysis Reveals MAPKKK Family Members Related to Drought Tolerance in Maize
Source: PLoS One. 2015 Nov 24;10(11):e0143128. doi: 10.1371/journal.pone.0143128 (PMC4658043; doi:10.1371/journal.pone.0143128)
Supplement: S1 File — contains five Supplementary tables: Table A. 71 predictive MAPKKKs in maize. Table B. The quality of the RNA-seq data for Q20 and Q30. Table C. RNA-seq data and mapping rates. Table D. The co-expressed MAPKKKs with DEGs in the enriched pathway. Table E. The common co-expressed MAPKKKs with our DEGs in the enriched pathway. (DOC) [file pone.0143128.s001.doc]

**RNA-seq analysis reveals MAPKKK family members related to drought tolerance in maize**

Ya Liu^1¶^, Miaoyi Zhou^1¶^, Zhaoxu Gao^2¶^, Wen Ren^1^, Fengling Yang^1^, Hang He^2*^, Jiuran Zhao^1*^

*^1^Maize Research Center, Beijing Academy of Agricultural and Forestry Science, Beijing 100097, P.R.China*

*^2^* *School of Life Sciences and School of Advanced Agriculture Sciences, Peking-Tsinghua Center for Life Sciences, Peking University, Beijing 100871, P.R.China*

^*^**Corresponding author**

**E-mail:** hehang@pku.edu.cn (HH)

maizezhao@126.com(JZ)

^¶^These authors contributed equally to this work.

# Supporting Information

Table A. 71 predictive MAPKKKs in maize. * indicate the eight differentially expressed MAPKKKs.

| Name | MAPKKKs_ID | Subcellular localization | Chr | subfamily | Predicted function(from WGS) |
| --- | --- | --- | --- | --- | --- |
| MAPKKK1 | GRMZM2G140726 | Nuclear | 10 | MEKK | hypothetical protein LOC100191147 |
| MAPKKK2 | GRMZM2G540772 | Nuclear | 5 | MEKK | unknown function |
| MAPKKK4 | GRMZM2G175504 | Nuclear | 2 | MEKK | unknown function |
| MAPKKK5 | GRMZM2G093316 | Nuclear | 4 | MEKK | hypothetical protein LOC100381765 |
| MAPKKK6 | AC209208.3 | Chloroplast | 5 | MEKK | unknown function |
| MAPKKK7 | GRMZM2G378479 | Extracellular | 2 | MEKK | LOC100281440 |
| MAPKKK8 | GRMZM2G034877 | Nuclear | 5 | MEKK | unknown function |
| MAPKKK9 | GRMZM2G156800 | Nuclear | 1 | MEKK | unknown function |
| MAPKKK10 | GRMZM2G180555 | Nuclear | 9 | MEKK | hypothetical protein LOC100383481 |
| MAPKKK11 | GRMZM2G066120 | Nuclear | 1 | MEKK | hypothetical protein LOC100272734 |
| MAPKKK12 | GRMZM2G130927 | Nuclear | 5 | MEKK | Putative uncharacterized protein |
| MAPKKK13 | GRMZM2G044557 | Chloroplast | 1 | MEKK | hypothetical protein LOC100279294 |
| MAPKKK15 | GRMZM2G064613 | Cytoplasmic | 4 | MEKK | hypothetical protein LOC100192646 |
| MAPKKK16 | GRMZM2G098828 | Nuclear | 2 | MEKK | unknown function |
| MAPKKK17 | GRMZM2G439350 | Chloroplast | 8 | MEKK | unknown function |
| MAPKKK18 | GRMZM2G305066* | Chloroplast | 8 | MEKK | unknown function |
| MAPKKK19 | GRMZM2G165099* | Chloroplast | 3 | MEKK | NPK1-related protein kinase-like protein Fragment |
| MAPKKK20 | GRMZM2G476477* | Chloroplast | 6 | MEKK | unknown function |
| MAPKKK21 | GRMZM2G173965* | Cytoplasmic | 8 | MEKK | unknown function |
| MAPKKK22 | GRMZM2G041774* | Chloroplast | 3 | MEKK | unknown function |
| MAPKKK23 | GRMZM2G116376 | Cytoplasmic | 4 | ZIK | serine/threonine-protein kinase WNK4 |
| MAPKKK24 | GRMZM2G878530 | Cytoplasmic | 5 | ZIK | WNK6 |
| MAPKKK26 | GRMZM2G021416* | Nuclear | 7 | ZIK | hypothetical protein LOC100383268 (LOC100383268), mRNA |
| MAPKKK28 | GRMZM2G312970 | Nuclear | 6 | ZIK | unknown function |
| MAPKKK29 | GRMZM2G011070 | Nuclear | Un | Raf | salt-inducible protein kinase |
| MAPKKK34 | GRMZM2G038982 | Nuclear | 1 | Raf | hypothetical protein LOC100275123 |
| MAPKKK35 | GRMZM2G175563 | Nuclear | 9 | Raf | hypothetical protein LOC100383170 |
| MAPKKK36 | GRMZM2G413069 | Cytoplasmic | 4 | Raf | unknown function |
| MAPKKK38 | GRMZM2G098187 | Chloroplast | 2 | Raf | unknown function |
| MAPKKK39 | GRMZM2G059671 | Chloroplast | 5 | Raf | hypothetical protein LOC100191496 |
| MAPKKK40 | GRMZM2G110572 | Nuclear | 1 | Raf | Putative uncharacterized protein |
| MAPKKK41 | GRMZM2G140537 | Nuclear | 3 | Raf | unknown function |
| MAPKKK42 | GRMZM2G448213 | Nuclear | 4 | Raf | unknown function |
| MAPKKK43 | GRMZM2G007854 | Nuclear | 4 | Raf | ATP binding protein |
| MAPKKK45 | GRMZM2G080499 | PlasmaMembrane | 3 | Raf | ATP binding protein |
| MAPKKK50 | GRMZM2G111269 | Cytoplasmic | 1 | Raf | hypothetical protein LOC100274496 |
| MAPKKK51 | GRMZM2G019434 | Mitochondrial | 1 | Raf | hypothetical protein LOC100192758 |
| MAPKKK52 | GRMZM2G131629 | Nuclear | 1 | Raf | unknown function |
| MAPKKK53 | GRMZM2G014618 | Nuclear | 5 | Raf | unknown function |
| MAPKKK54 | GRMZM2G063684 | Cytoplasmic | 8 | Raf | hypothetical protein LOC100381637 |
| MAPKKK55 | GRMZM2G088299 | Nuclear | 3 | Raf | hypothetical protein LOC100194330 |
| MAPKKK56 | GRMZM2G063069* | Cytoplasmic | 8 | Raf | ATP binding protein |
| MAPKKK57 | GRMZM2G814851 | Cytoplasmic | 7 | Raf | ATP binding protein |
| MAPKKK59 | GRMZM2G459854 | Cytoplasmic | 2 | Raf | hypothetical protein LOC100382522 |
| MAPKKK60 | GRMZM2G164242 | Cytoplasmic | 5 | Raf | unknown function |
| MAPKKK61 | GRMZM2G160922 | Cytoplasmic | 7 | Raf | hypothetical protein LOC100382036 |
| MAPKKK63 | GRMZM2G152889 | Cytoplasmic | 3 | Raf | ATP binding protein (LOC100285203), mRNA |
| MAPKKK64 | GRMZM2G156013 | Chloroplast | 10 | Raf | serine/threonine protein kinase |
| MAPKKK65 | GRMZM2G102088 | Cytoplasmic | 2 | Raf | serine/threonine protein kinase |
| MAPKKK66 | GRMZM2G140612 | Cytoplasmic | 10 | Raf | LOC100282546 |
| MAPKKK67 | GRMZM2G028604 | Nuclear | 9 | Raf | HT1 protein kinase |
| MAPKKK68 | GRMZM2G018280 | Nuclear | 6 | Raf | hypothetical protein LOC100381973 (LOC100381973), mRNA |
| MAPKKK69 | GRMZM2G171677 | Nuclear | 3 | Raf | hypothetical protein LOC100191726 |
| MAPKKK70 | GRMZM2G097878 | Nuclear | 8 | Raf | hypothetical protein LOC100279762 |
| MAPKKK72 | GRMZM2G114093 | Nuclear | 1 | Raf | protein kinase |
| MAPKKK73 | GRMZM2G474546* | Nuclear | 6 | Raf | hypothetical protein LOC100193102 |
| MAPKKK74 | GRMZM2G104283 | Nuclear | 8 | Raf | protein kinase |
| MAPKKK75 | GRMZM2G459824 | Extracellular | 1 | MEKK | unknown function |
| MAPKKK76 | GRMZM2G404078 | Chloroplast | 3 | MEKK | unknown function |
| MAPKKK77 | GRMZM2G335826 | Chloroplast | 3 | MEKK | unknown function |
| MAPKKK78 | GRMZM2G158860 | Mitochondrial | 4 | MEKK | hypothetical protein LOC100275358 |
| MAPKKK79 | AC204050.4 | Chloroplast | 6 | MEKK | unknown function |
| MAPKKK80 | GRMZM2G378852 | Chloroplast | 9 | MEKK | unknown function |
| MAPKKK81 | GRMZM2G032619 | Nuclear | 4 | ZIK | unknown function |
| MAPKKK82 | GRMZM2G034779 | Nuclear | 7 | ZIK | unknown function |
| MAPKKK83 | GRMZM2G023444 | Nuclear | 7 | ZIK | hypothetical protein LOC100273338 |
| MAPKKK84 | GRMZM2G072395 | Nuclear | 8 | ZIK | hypothetical protein LOC100216969 |
| MAPKKK85 | GRMZM2G002531 | Cytoplasmic | 4 | Raf | ATP binding protein |
| MAPKKK86 | GRMZM2G028709 | Nuclear | 4 | Raf | serine/threonine-protein kinase CTR1 |
| MAPKKK87 | GRMZM2G852329 | Chloroplast | 4 | Raf | Putative uncharacterized protein |
| MAPKKK88 | GRMZM2G127632 | Nuclear | 5 | Raf | unknown function |

Table B.The quality of the RNA-seq data for Q20 and Q30.( Q20 and Q30 represents for the percentage of the base whose quality is equal to 20 or 30. )

| Sample | Q20 | Q30 |
| --- | --- | --- |
| Leaf-co | 98.53% | 87.12% |
| Leaf-dr | 98.59% | 87.65% |
| Stem-co | 99.38% | 94.15% |
| Stem-dr | 99.34% | 93.85% |
| Root-co | 99.37% | 94.06% |
| Root-dr | 99.36% | 93.97% |

Table C. RNA-seq data and mapping rates.

| Sample | Total reads | Aligned reads | Mapping rates (%) |
| --- | --- | --- | --- |
| Leaf-co | 20,862,719 | 18,084,389 | 86.68 |
| Stem-co | 20,521,909 | 17,051,719 | 83.09 |
| Root-co | 20,395,835 | 17,785,168 | 87.20 |
| Leaf-dr | 18,737,440 | 14,876,284 | 79.39 |
| Stem-dr | 27,357,572 | 22,317,412 | 81.58 |
| Root-dr | 25,206,868 | 18,219,524 | 72.28 |

Table D. The co-expressed MAPKKKs with DEGs in the enriched pathway

| pathway | MAPKKKs | Z-score |
| --- | --- | --- |
| Oxidate | MAPKKK1 MAPKKK4 MAPKKK5 MAPKKK7 MAPKKK8 MAPKKK9 | 4.7946 |
|  | MAPKKK11 MAPKKK12 MAPKKK13 MAPKKK15 MAPKKK17 MAPKKK19 |  |
|  | MAPKKK21 MAPKKK22 MAPKKK34 MAPKKK36 MAPKKK38 MAPKKK39 |  |
|  | MAPKKK43 MAPKKK51 MAPKKK54 MAPKKK55 MAPKKK56 MAPKKK63 |  |
|  | MAPKKK64 MAPKKK65 MAPKKK69 MAPKKK72 MAPKKK73 MAPKKK74 |  |
|  | MAPKKK76 MAPKKK78 MAPKKK81 MAPKKK83 MAPKKK85 MAPKKK86 |  |
|  | MAPKKK88 |  |
| Photosynothesis | MAPKKK1 MAPKKK8 MAPKKK9 MAPKKK11 MAPKKK12 MAPKKK13 | 5.2704 |
|  | MAPKKK15 MAPKKK22 MAPKKK26 MAPKKK34 MAPKKK36 MAPKKK38 |  |
|  | MAPKKK39 MAPKKK51 MAPKKK56 MAPKKK63 MAPKKK65 MAPKKK72 |  |
|  | MAPKKK73 MAPKKK74 MAPKKK83 MAPKKK86 MAPKKK88 |  |
| Auxin | MAPKKK5 MAPKKK8 MAPKKK9 MAPKKK11 MAPKKK12 MAPKKK13 | 3.8115 |
|  | MAPKKK15 MAPKKK17 MAPKKK19 MAPKKK21 MAPKKK22 MAPKKK34 |  |
|  | MAPKKK35 MAPKKK36 MAPKKK38 MAPKKK39 MAPKKK40 MAPKKK41 |  |
|  | MAPKKK50 MAPKKK51 MAPKKK54 MAPKKK55 MAPKKK56 MAPKKK61 |  |
|  | MAPKKK63 MAPKKK64 MAPKKK65 MAPKKK69 MAPKKK72 MAPKKK73 |  |
|  | MAPKKK74 MAPKKK76 MAPKKK78 MAPKKK81 MAPKKK83 MAPKKK86 |  |
|  | MAPKKK88 |  |
| Cytokinine | MAPKKK9 MAPKKK11 MAPKKK12 MAPKKK13 MAPKKK15 MAPKKK19 | 4.5556 |
|  | MAPKKK21 MAPKKK22 MAPKKK34 MAPKKK35 MAPKKK38 MAPKKK39 |  |
|  | MAPKKK40 MAPKKK45 MAPKKK51 MAPKKK54 MAPKKK56 MAPKKK61 |  |
|  | MAPKKK63 MAPKKK64 MAPKKK65 MAPKKK69 MAPKKK72 MAPKKK74 |  |
|  | MAPKKK78 MAPKKK83 MAPKKK86 MAPKKK88 |  |
| GA | MAPKKK9 MAPKKK11 MAPKKK12 MAPKKK13 MAPKKK19 MAPKKK22 | 2.5264 |
|  | MAPKKK34 MAPKKK38 MAPKKK39 MAPKKK51 MAPKKK56 MAPKKK63 |  |
|  | MAPKKK64 MAPKKK65 MAPKKK69 MAPKKK72 MAPKKK74 MAPKKK78 |  |
|  | MAPKKK83 MAPKKK88 |  |
| ABA | MAPKKK8 MAPKKK11 MAPKKK12 MAPKKK13 MAPKKK22 MAPKKK34 | 4.3080 |
|  | MAPKKK38 MAPKKK39 MAPKKK43 MAPKKK51 MAPKKK56 MAPKKK65 |  |
|  | MAPKKK72 MAPKKK74 MAPKKK83 MAPKKK88 |  |
| Enthylene | MAPKKK8 MAPKKK9 MAPKKK11 MAPKKK12 MAPKKK13 MAPKKK17 | 2.6170 |
|  | MAPKKK19 MAPKKK21 MAPKKK22 MAPKKK34 MAPKKK36 MAPKKK38 |  |
|  | MAPKKK39 MAPKKK51 MAPKKK56 MAPKKK63 MAPKKK64 MAPKKK65 |  |
|  | MAPKKK69 MAPKKK72 MAPKKK74 MAPKKK76 MAPKKK78 MAPKKK81 |  |
|  | MAPKKK83 MAPKKK88 |  |
| BR | MAPKKK8 MAPKKK9 MAPKKK11 MAPKKK12 MAPKKK13 MAPKKK15 | 2.8939 |
|  | MAPKKK21 MAPKKK22 MAPKKK34 MAPKKK38 MAPKKK39 MAPKKK51 |  |
|  | MAPKKK56 MAPKKK63 MAPKKK64 MAPKKK65 MAPKKK72 MAPKKK74 |  |
|  | MAPKKK76 MAPKKK83 |  |
| Jasmonic | MAPKKK9 MAPKKK11 MAPKKK12 MAPKKK13 MAPKKK17 MAPKKK19 | 2.8655 |
|  | MAPKKK21 MAPKKK22 MAPKKK34 MAPKKK36 MAPKKK38 MAPKKK39 |  |
|  | MAPKKK51 MAPKKK56 MAPKKK61 MAPKKK63 MAPKKK64 MAPKKK65 |  |
|  | MAPKKK69 MAPKKK72 MAPKKK74 MAPKKK76 MAPKKK78 MAPKKK83 |  |
|  | MAPKKK88 |  |
| Salicylic | MAPKKK4 MAPKKK9 MAPKKK11 MAPKKK12 MAPKKK13 MAPKKK15 | 3.4308 |
|  | MAPKKK17 MAPKKK19 MAPKKK21 MAPKKK22 MAPKKK34 MAPKKK36 |  |
|  | MAPKKK38 MAPKKK39 MAPKKK51 MAPKKK56 MAPKKK63 MAPKKK64 |  |
|  | MAPKKK65 MAPKKK69 MAPKKK72 MAPKKK74 MAPKKK76 MAPKKK78 |  |
|  | MAPKKK83 MAPKKK86 MAPKKK88 |  |
| Starch | MAPKKK1 MAPKKK7 MAPKKK8 MAPKKK9 MAPKKK11 MAPKKK12 | 4.2645 |
|  | MAPKKK13 MAPKKK17 MAPKKK19 MAPKKK21 MAPKKK22 MAPKKK34 |  |
|  | MAPKKK36 MAPKKK38 MAPKKK39 MAPKKK43 MAPKKK51 MAPKKK54 |  |
|  | MAPKKK55 MAPKKK56 MAPKKK61 MAPKKK63 MAPKKK64 MAPKKK65 |  |
|  | MAPKKK69 MAPKKK72 MAPKKK74 MAPKKK76 MAPKKK78 MAPKKK81 |  |
|  | MAPKKK83 MAPKKK86 MAPKKK88 |  |
| Spliceosome | MAPKKK1 MAPKKK4 MAPKKK5 MAPKKK7 MAPKKK8 MAPKKK9 | 5.3866 |
|  | MAPKKK10 MAPKKK11 MAPKKK12 MAPKKK13 MAPKKK15 MAPKKK17 |  |
|  | MAPKKK19 MAPKKK21 MAPKKK22 MAPKKK28 MAPKKK34 MAPKKK35 |  |
|  | MAPKKK36 MAPKKK38 MAPKKK39 MAPKKK40 MAPKKK41 MAPKKK43 |  |
|  | MAPKKK50 MAPKKK51 MAPKKK54 MAPKKK55 MAPKKK56 MAPKKK58 |  |
|  | MAPKKK63 MAPKKK64 MAPKKK65 MAPKKK69 MAPKKK72 MAPKKK74 |  |
|  | MAPKKK76 MAPKKK78 MAPKKK81 MAPKKK83 MAPKKK85 MAPKKK88 |  |
| Arginine and proline | MAPKKK1 MAPKKK5 MAPKKK8 MAPKKK9 MAPKKK11 MAPKKK12 | 3.6901 |
|  | MAPKKK13 MAPKKK15 MAPKKK17 MAPKKK19 MAPKKK21 MAPKKK22 |  |
|  | MAPKKK34 MAPKKK36 MAPKKK38 MAPKKK39 MAPKKK40 MAPKKK41 |  |
|  | MAPKKK51 MAPKKK54 MAPKKK55 MAPKKK56 MAPKKK63 MAPKKK64 |  |
|  | MAPKKK65 MAPKKK69 MAPKKK72 MAPKKK74 MAPKKK76 MAPKKK78 |  |
|  | MAPKKK81 MAPKKK83 MAPKKK86 MAPKKK88 |  |
| TCA cycle | MAPKKK4 MAPKKK5 MAPKKK8 MAPKKK9 MAPKKK11 MAPKKK12 | 4.6167 |
|  | MAPKKK13 MAPKKK15 MAPKKK17 MAPKKK19 MAPKKK21 MAPKKK22 |  |
|  | MAPKKK34 MAPKKK36 MAPKKK38 MAPKKK39 MAPKKK51 MAPKKK54 |  |
|  | MAPKKK56 MAPKKK63 MAPKKK64 MAPKKK65 MAPKKK69 MAPKKK72 |  |
|  | MAPKKK74 MAPKKK76 MAPKKK78 MAPKKK81 MAPKKK83 MAPKKK86 |  |
|  | MAPKKK88 |  |
| Phenylalanine | MAPKKK1 MAPKKK5 MAPKKK7 MAPKKK8 MAPKKK9 MAPKKK11 | 0.6680 |
|  | MAPKKK12 MAPKKK13 MAPKKK15 MAPKKK17 MAPKKK19 MAPKKK21 |  |
|  | MAPKKK22 MAPKKK26 MAPKKK34 MAPKKK35 MAPKKK36 MAPKKK38 |  |
|  | MAPKKK39 MAPKKK40 MAPKKK43 MAPKKK45 MAPKKK50 MAPKKK51 |  |
|  | MAPKKK54 MAPKKK55 MAPKKK56 MAPKKK60 MAPKKK63 MAPKKK64 |  |
|  | MAPKKK65 MAPKKK66 MAPKKK69 MAPKKK72 MAPKKK73 MAPKKK74 |  |
|  | MAPKKK76 MAPKKK78 MAPKKK81 MAPKKK82 MAPKKK83 MAPKKK85 |  |
|  | MAPKKK86 MAPKKK88 |  |
| Phosphatidylinositol | MAPKKK1 MAPKKK8 MAPKKK9 MAPKKK11 MAPKKK12 MAPKKK13 | 4.1057 |
|  | MAPKKK15 MAPKKK17 MAPKKK19 MAPKKK21 MAPKKK22 MAPKKK34 |  |
|  | MAPKKK36 MAPKKK38 MAPKKK39 MAPKKK41 MAPKKK45 MAPKKK51 |  |
|  | MAPKKK54 MAPKKK56 MAPKKK63 MAPKKK64 MAPKKK65 MAPKKK69 |  |
|  | MAPKKK72 MAPKKK74 MAPKKK76 MAPKKK78 MAPKKK83 MAPKKK85 |  |
|  | MAPKKK86 MAPKKK88 |  |
| RNA polymerase | MAPKKK4 MAPKKK5 MAPKKK8 MAPKKK9 MAPKKK10 MAPKKK11 | 3.5227 |
|  | MAPKKK12 MAPKKK13 MAPKKK15 MAPKKK17 MAPKKK19 MAPKKK21 |  |
|  | MAPKKK22 MAPKKK34 MAPKKK35 MAPKKK36 MAPKKK38 MAPKKK39 |  |
|  | MAPKKK40 MAPKKK43 MAPKKK51 MAPKKK54 MAPKKK56 MAPKKK63 |  |
|  | MAPKKK64 MAPKKK65 MAPKKK69 MAPKKK72 MAPKKK74 MAPKKK76 |  |
|  | MAPKKK78 MAPKKK83 MAPKKK88 |  |
| CoA | MAPKKK9 MAPKKK11 MAPKKK12 MAPKKK13 MAPKKK15 MAPKKK17 | 5.3771 |
|  | MAPKKK19 MAPKKK21 MAPKKK22 MAPKKK34 MAPKKK36 MAPKKK38 |  |
|  | MAPKKK39 MAPKKK41 MAPKKK51 MAPKKK56 MAPKKK63 MAPKKK64 |  |
|  | MAPKKK65 MAPKKK69 MAPKKK72 MAPKKK74 MAPKKK76 MAPKKK78 |  |
|  | MAPKKK83 MAPKKK85 MAPKKK88 |  |
| Circadian | MAPKKK9 MAPKKK11 MAPKKK12 MAPKKK13 MAPKKK15 MAPKKK17 | 5.2424 |
|  | MAPKKK19 MAPKKK21 MAPKKK22 MAPKKK34 MAPKKK36 MAPKKK38 |  |
|  | MAPKKK39 MAPKKK50 MAPKKK51 MAPKKK55 MAPKKK56 MAPKKK63 |  |
|  | MAPKKK64 MAPKKK65 MAPKKK69 MAPKKK72 MAPKKK74 MAPKKK76 |  |
|  | MAPKKK78 MAPKKK83 MAPKKK86 MAPKKK88 |  |

Table E. The common co-expressed MAPKKKs with our DEGs in the enriched pathway

| gene | log2(fold_change)-leaf | log2(fold_change)-stem | log2(fold_change)-root |
| --- | --- | --- | --- |
| MAPKKK11 | 0.2021 | -0.4573 | 1.0869 |
| MAPKKK12 | 0.4686 | -0.1034 | 0.7059 |
| MAPKKK13 | -0.1747 | -0.1531 | 1.2276 |
| MAPKKK22 | 0.3924 | 3.4326 | 1.849 |
| MAPKKK34 | 0.0439 | -0.2603 | 0.4587 |
| MAPKKK38 | -0.2858 | -0.2503 | 1.6345 |
| MAPKKK39 | -0.4266 | 0.0187 | 1.5017 |
| MAPKKK51 | -0.1004 | 0.3204 | 0.3629 |
| MAPKKK56 | 2.9552 | 0.1292 | 1.1927 |
| MAPKKK65 | -0.1987 | 0.0435 | 0.7108 |
| MAPKKK72 | -0.4332 | -0.6036 | 0.9351 |
| MAPKKK74 | 0.0846 | -0.0786 | 1.0012 |
| MAPKKK83 | -0.5683 | 0.9334 | 2.5268 |
|  |  |  |  |
